# Electrode reconstruction strategy for oxygen evolution reaction: maintaining Fe-CoOOH phase with intermediate-spin state during electrolysis

**DOI:** 10.1038/s41467-022-28260-5

**Published:** 2022-02-01

**Authors:** Woong Hee Lee, Man Ho Han, Young-Jin Ko, Byoung Koun Min, Keun Hwa Chae, Hyung-Suk Oh

**Affiliations:** 1 Clean Energy Research Center, Korea Institute of Science and Technology (KIST), Hwarang-ro 14-gil 5, Seongbuk-gu, Seoul, 02792 Republic of Korea; 2 Department of Chemistry, Seoul National University, Seoul, 08826 Republic of Korea; 3 Department of Chemical and Biological Engineering, Korea University, Anamdong-5-Ga, Seoul, 02841 Republic of Korea; 4 Graduate School of Energy and Environment (KU-KIST Green School), Korea University, 145 Anam-ro, Seongbuk-gu, Seoul, 02841 Republic of Korea; 5 Advanced Analysis Center, Korea Institute of Science and Technology (KIST), Hwarang-ro 14-gil 5, Seongbuk-gu, Seoul, 02792 Republic of Korea; 6 Division of Energy and Environmental Technology, KIST school, Korea University of Science and Technology, Seoul, 02792 Republic of Korea; 7 KIST-SKKU Carbon-Neutral Research Center, Sungkyunkwan University, 2066 Seobu-ro, Jangan-gu, Suwon, 16419 Republic of Korea

**Keywords:** Electrocatalysis, Electrocatalysis, Nanoscale materials, Catalytic mechanisms

## Abstract

Computational calculations and experimental studies reveal that the CoOOH phase and the intermediate-spin (IS) state are the key factors for realizing efficient Co-based electrocatalysts for the oxygen evolution reaction (OER). However, according to thermodynamics, general cobalt oxide converts to the CoO_2_ phase under OER condition, retarding the OER kinetics. Herein, we demonstrate a simple and scalable strategy to fabricate electrodes with maintaining Fe-CoOOH phase and an IS state under the OER. The changes of phase and spin states were uncovered by combining in-situ*/operando* X-ray based absorption spectroscopy and Raman spectroscopy. Electrochemical reconstruction of chalcogenide treated Co foam affords a highly enlarged active surface that conferred excellent catalytic activity and stability in a large-scale water electrolyzer. Our findings are meaningful in that the calculated results were experimentally verified through the *operando* analyses. It also proposes a new strategy for electrode fabrication and confirms the importance of real active phases and spin states under a particular reaction condition.

## Abstract (web summary)

The phase and spin state affect catalytic activity of Co-based catalysts for oxygen evolution reaction. Herein, the authors demonstrate a simple reconstruction strategy to fabricate electrodes maintaining a Fe-CoOOH phase and an intermediate-spin state during catalysis.

## Introduction

Water electrolysis is a clean and useful technology for producing hydrogen, which is a renewable alternative to fossil energies^1,2^. However, the oxygen evolution reaction (OER) is a bottleneck in realizing efficient water electrolysis owing to its sluggish kinetics^3–6^. The use of noble metal-based catalysts such as Ir and Ru oxides can accelerate the OER kinetics^7,8^. However, the high cost and scarcity of noble metals limit their industrial application in water electrolysis. Thus, significant computational and experimental studies have been conducted to develop efficient and cost-effective OER catalysts with earth-abundant metals.

In this regard, Co-based catalysts have attractive significant attention for application in alkaline OER owing to their high OER activity, low cost, and the mixed oxidation state, which enables the generation of various Co-based materials. Various cobalt compounds such as CoO, Co_3_O_4_, Co_4_O_4_, cubane, cobalt hydroxide, cobalt oxyhydroxide, perovskite, cobalt phosphide, cobalt borate, and cobalt sulfide have been reported to be promising OER catalysts^9–23^. Many studies have focused on analyzing the properties of the pristine catalysts and have conducted density functional theory (DFT) calculations using the initial state of the catalysts. However, the initial phase of the Co-based catalyst may transform under alkaline OER conditions. For example, cobalt hydroxide is thermodynamically oxidized under alkaline OER conditions as follows^24,25^:

$$3{{{\rm{Co}}}}{\left({{{\rm{OH}}}}\right)}_{2}+\,2{{{{\rm{OH}}}}}^{-}\leftrightarrow \,{{{{\rm{Co}}}}}_{3}{{{{\rm{O}}}}}_{4}+\,4{{{{\rm{H}}}}}_{2}{{{\rm{O}}}}+2{{{{\rm{e}}}}}^{-}\,(0.81\,{{{{\rm{V}}}}}_{{{{\rm{RHE}}}}})$$

$${{{{\rm{Co}}}}}_{3}{{{{\rm{O}}}}}_{4}+\,{{{{\rm{OH}}}}}^{-}+\,{{{{\rm{H}}}}}_{2}{{{\rm{O}}}}\leftrightarrow \,3{{{\rm{CoOOH}}}}+{{{{\rm{e}}}}}^{-}\,(1.22\,{{{{\rm{V}}}}}_{{{{\rm{RHE}}}}})$$

$${{{\rm{CoOOH}}}}+\,{{{{\rm{OH}}}}}^{-}\leftrightarrow {{{\rm{Co}}}}{{{{\rm{O}}}}}_{2}+\,{{{{\rm{H}}}}}_{2}{{{\rm{O}}}}+{{{{\rm{e}}}}}^{-}\,(1.56\,{{{{\rm{V}}}}}_{{{{\rm{RHE}}}}})$$

The transformation of the cobalt catalyst indicates that the OER activity of the catalyst is strongly dependent on the converted final phase of cobalt during the OER rather than its initial phase. Therefore, it is significantly important to identify the real phase of the Co-based catalysts during the OER, and this can be achieved using various in-situ*/operando* spectroscopic techniques. The transformation of the Co phase from CoO_2_ to CoOOH and from CoOOH to CoO_x_ (predicted to be Co(VI), CoO_2_) under OER conditions has already been reported using in-situ*/operando* Raman spectroscopy, suggesting that the real phase of Co-based catalysts for OER is CoO_2_^26–28^. This observation was consistent with the predictions based on electrochemical thermodynamics. However, using time-resolved Fourier transform-infra-red (FT-IR) spectroscopy, Frei et al. found that the OER kinetics are delayed more in the presence of Co(IV)=O species than in the presence of Co(III)OH species^29^. Strasser group also found that Co^3+^ species in CoO_x_(OH)_y_ play an important role for high OER activity by acting as a fast active site^30,31^. According to the recently reported Co-based catalysts with excellent OER catalytic activity, the initial different types of catalysts are converted to similar CoOOH phases under OER conditions^32^. Therefore, it is important to identify the active sites during actual OER and design the electrode with Co(III)OH species with high catalytic activity without converting to Co(IV)=O species. Furthermore, recent studies have revealed that not only the phase of cobalt but also its spin state affects the OER activity of Co-based catalysts^33–37^. The *e*_*g*_ occupancy determined by the spin state is strongly affects the binding of oxygen. Shao-Horn and coworkers reported that the spin states of cobalt and *e*_*g*_ occupancy significantly influences the binding strength of the oxygen intermediates^37^. According to various DFT calculations, the intermediate spin (IS, *t*_*2g*_^5^*e*_*g*_^1^) of Co^3+^ has ideal *e*_*g*_ occupancy, which is expected to confer excellent OER performance. Thus, fabricating Co electrodes with an appropriate CoOOH phase and IS state for the OER is a key to the practical implementation of alkaline water electrolysis.

Here, we proposed a simple and scalable strategy to convert a cobalt foam (CF) electrode into a highly active catalyst for the OER through sulfur and iron treatment with the goal of maintaining the CoOOH phase and IS state of the Co-based electrode under OER conditions. The surface of the modified CF electrode was reconstructed to accommodate the Fe-CoOOH species under alkaline conditions. With increasing applied potential, the Fe-CoOOH species in the modified CF electrode was retained and not transformed to CoO_2_. This was observed using in-situ*/operando* Raman spectroscopy and X-ray absorption fine spectroscopy (XAFS). In addition, changes in the spin state of the Co-based electrode under OER conditions were observed through in-situ*/operando* near edge X-ray absorption fine structure (NEXAFS). The prepared CF electrode underwent conversion from the low spin (LS) state to IS under OER conditions and remained in this state. These experimental *operando* analyses could explain the improved OER activity of the prepared CF electrode. The synthetic method and *operando* analysis presented herein are a viable route to design and implement oxidation electrodes for large-scale electrolysis systems.

## Results

### Synthesis and morphology of the Fe-CoOOH electrode during OER

**Fig. 1:**
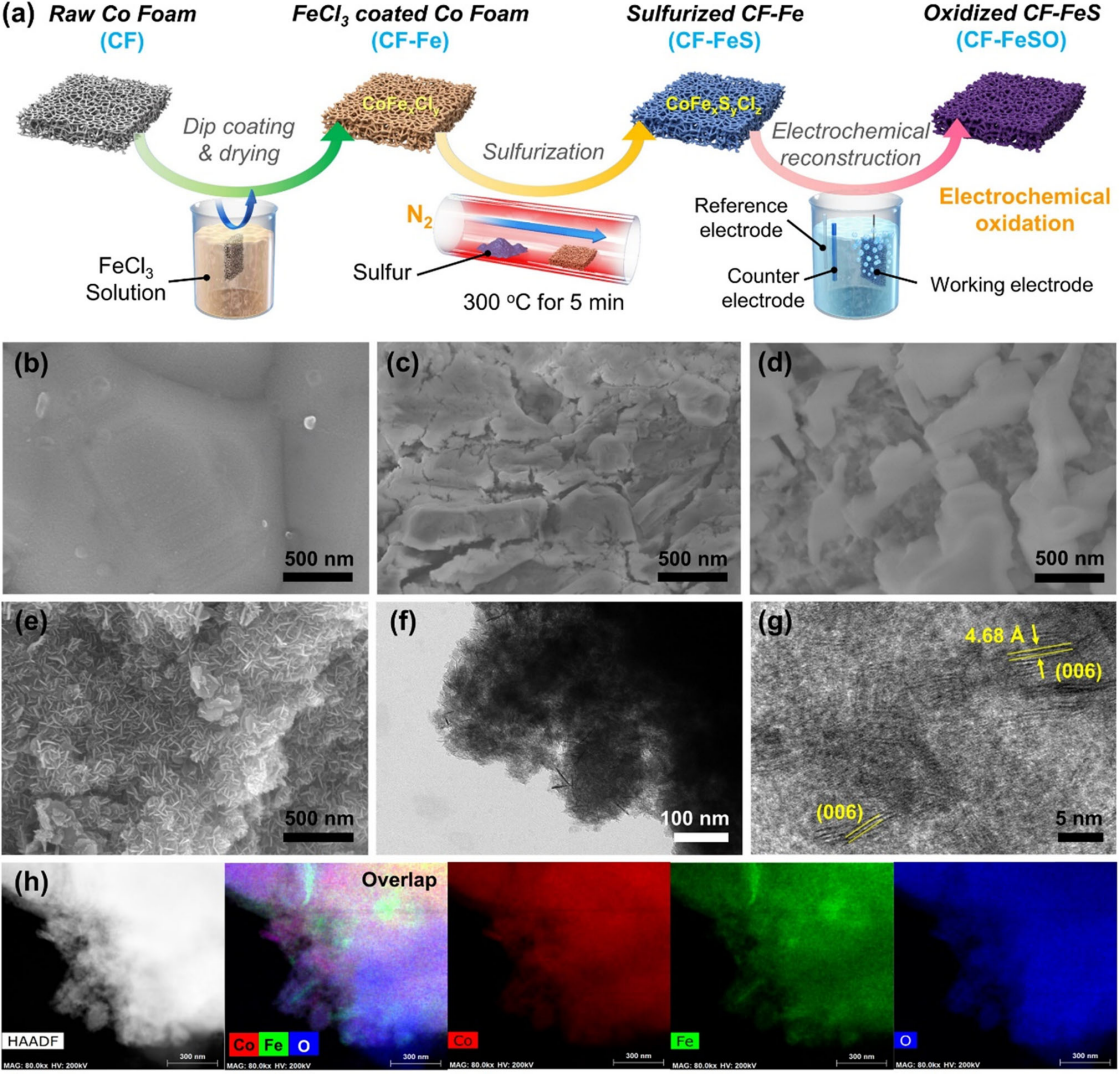
Fabrication, morphology, and elemental mapping of the Fe-CoOOH electrode. **a** Schematic of the fabrication of the sulfurized CoFe electrode after electrochemical oxidation (denoted as CF-FeSO). SEM images of the electrode at each step during the fabrication of the CF-FeSO electrode: **b** Raw CF, **c** CF-Fe, **d** CF-FeS, and **e** CF-FeSO. **f**, **g** HR-TEM images of CF-FeSO, **h** Energy dispersive X-ray spectroscopy (EDS) elemental maps of CF-FeSO using TEM.

The Fe-CoOOH electrode for the OER was fabricated by the surface reconstruction strategy under alkaline OER conditions by treating the raw CF with iron and sulfur (Fig. 1a). To verify the effect of S and Fe treatment, the CF after OER (CF-O) and sulfur-treated CF after OER (CF-SO) were also prepared. For the Fe treatment, the CF was subjected to dip coating by soaking the CF (Fig. 1b) in 0.25 M FeCl_3_ solution for 30 s and then drying in an oven at 70 °C for 1 h^38–40^. The Fe treatment (CF-Fe) led to corrosion and cracks on the CF owing to Cl ion (Fig. 1c). The CF-Fe surface was oxidized and converted to CoFe_x_Cl_y_ (Supplementary Figs. 2–4). Next, for the sulfur treatment, CF-Fe was thermally treated with S powder at 300 °C for 5 min in a furnace. During the sulfur treatment, some of the S anions reacted with cobalt and iron of CF-Fe while the other S anions replaced the Cl^‒^ anions of CF-Fe (Fig. 1d, Supplementary Figs. 4 and 5). Co and Fe of sulfur-treated CF-Fe (CF-FeS) were softly reduced compared to CF-Fe due to the lower electronegativity of sulfur than that of chlorine (Supplementary Fig. 2). X-ray photoelectron spectroscopy (XPS) spectrum suggested that Co and S were present at an atomic ratio of 1:1.74. After the S and Fe treatment, electrochemical reconstruction was performed under OER conditions to produce CF-FeSO. The S and remaining Cl^‒^ species were dissolved and exchanged with oxygen species (O^2‒^, OH^‒^), thereby reconstructing the highly rough structure with abundant Fe-doped Co nanosheets (Fig. 1e, Supplementary Figs. 4 and 5). The reconstruction under OER conditions oxidized more CF-FeS surface, leading to the generation of abundant Co^3+^ species and Fe^3+^ species in CF-FeSO (Supplementary Figs. 2–5). Scanning electron microscopy (SEM) images in Fig. 1e and Supplementary Fig. 6 revealed that CF-FeSO possesses abundant nanosheet structures on the highly rough surface. CF-O has large Co nanosheets on the plain surface (Supplementary Fig. 7), while the surface of CF-SO is highly rough, with grown abundant Co nanosheet layers which were smaller than those in CF-O (Supplementary Fig. 8). Transmission electron microscopy (TEM) images of CF-FeSO showed the formation of thin nanosheets with an oxidized structure on the CF surface, such as layered double hydroxide structure (Fig. 1f, g). Energy dispersive X-ray spectroscopy (EDS) of the TEM and SEM images reveals the homogenous incorporation of Fe on the CF despite the rough dip coating process (Fig. 1h and Supplementary Fig. 9). As shown in the EDS element mapping result (Supplementary Fig. 10), compared to the inside of CF-FeSO, the element ratio of S and Cl on the surface decreased while the ratio of O increased, confirming that S and Cl species were exchanged with O species. Thus, this easy and scalable process successfully converted raw CF into not only a substrate of the electrode but also into a catalytically active site with a large surface area. Furthermore, the electrochemical reconstruction strategy could be adopted for cobalt oxide nanoparticles (CPs) instead of CF. The X-ray diffraction (XRD) patterns and TEM images of CP-FeSO indicate well reconstructed structure, suggesting the high flexibility of this strategy (Supplementary Figs. 11 and 12).

### Characterization of fabricated CF electrode

**Fig. 2:**
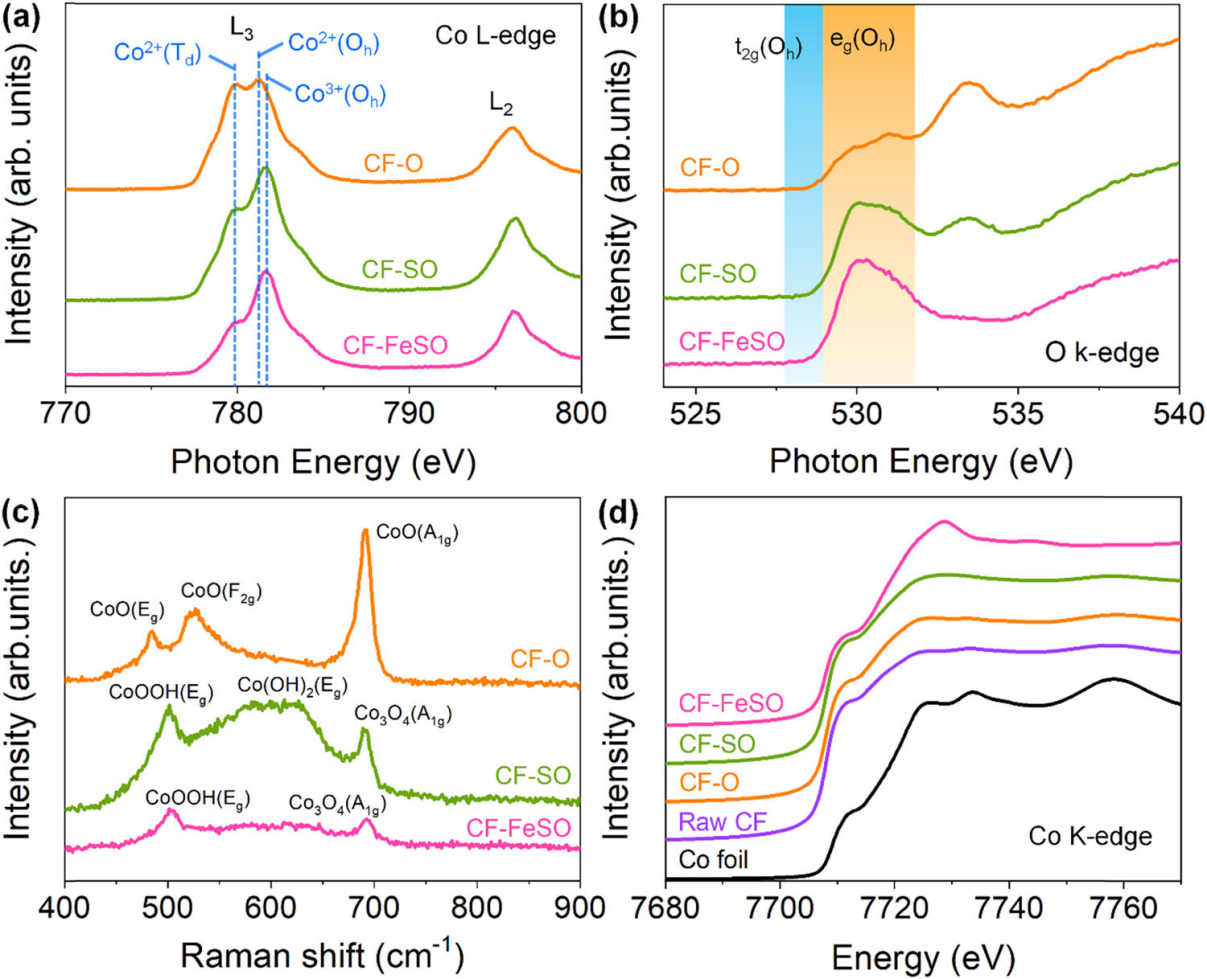
Electronic structure of the CF-based electrodes. **a** Co L-edge near edge X-ray absorption fine structure (NEXAFS) spectra of CF-O, CF-SO and, CF-FeSO. **b** O K-edge NEXAFS spectra of CF-O, CF-SO and, CF-FeSO. **c** Raman spectra of CF-O, CF-SO and, CF-FeSO. **d** Co K-edge XANES of Co foil, Raw CF, CF-O, CF-SO and, CF-FeSO.

To better understand the formation of CF-FeSO, surface-sensitive characterization techniques such as NEXAFS spectroscopy and Raman spectroscopy were employed (Fig. 2a, b, c). The Co L-edge of CF-O exhibits peaks corresponding to Co^2+^(T_d_) and Co^2+^(O_h_) with the 3d^7^ configuration (Fig. 2a), indicating that the CF-O surface contains CoO^41^. This electronic structure can also be inferred from the O K-edge NEXAFS spectrum, which represents the hybridization of the Co 3*d* and O 2*p* orbitals^42,43^. The configuration of unfilled t_2g_ orbitals and the extremely small hole of the *e*_*g*_ orbitals of CF-O leads small peak a t 530 eV, which could be related to the high spin (HS) state of the Co^2+^-*t*_*2g*_^*5*^*e*_*g*_^*2*^ orbitals (Fig. 2b). CF-SO possess Co^3+^(O_h_) instead of Co^2+^(O_h_), suggesting that the ratio of Co^3+^(O_h_) and Co^2+^(T_d_) is 1.56:1. Co_3_O_4_ has a spinel structure, i.e., Co^2+^(T_d_)_1_ Co^3+^(O_h_)_2_O_4_. Thus, the state of CF-SO is between CoO and Co_3_O_4_. The O K-edge of CF-SO shows no t_2g_ peak and more intense e_g_ orbital peaks than that of CF-O owing to the presence of low spin Co^3+^(O_h_) with t_2g_^6^e_g_^0^ orbital configuration. The Co L-edge of CF-FeSO demonstrates more intense Co^3+^(O_h_) peaks than that of CF-SO, suggesting substantial contribution from the 3d^6^ configuration. The ratio of Co^3+^(O_h_) and Co^2+^(T_d_) was 2.32:1, indicating that CF-FeSO is a mixture of CoOOH and Co_3_O_4_. The only *e*_*g*_ orbital peaks of CF-FeSO were observed in the O K-edge, suggesting completely filled *t*_*2g*_ orbitals. This suggests that CoOOH is present in the LS (*t*_*2g*_^*6*^*e*_*g*_^*0*^) state in CF-FeSO. The Raman spectra further confirm the phase of the CF-based electrode (Fig. 2c). The Raman spectrum of CF-O exhibits three peaks corresponding to E_g_, F_2g_, and A_1g_, revealing the CoO phase^44^. The spectrum of CF-SO also shows three peaks corresponding to E_g_ of CoOOH, E_g_ of Co(OH)_2_, and A_1g_ of Co_3_O_4_, indicating a mixture of Co(OH)_2_, Co_3_O_4_, and CoOOH. Two peaks, corresponding to E_g_ of CoOOH and A_1g_ of Co_3_O_4_, were observed in the spectrum of CF-FeSO^45^.

To observe the entire Co electronic state in the CF-based electrode, Co K-edge X-ray absorption near edge structure (XANES) spectroscopy, which is a bulk-sensitive technique, was performed (Fig. 2d). The spectrum of raw CF, which has an almost metallic character, was different from that of a Co foil because of the self-absorption due to the thick Co foam. Generally, the pre-edge peak (1 *s* → 3*d* for Co) is more accurate for estimating the oxidation state. However, a huge amount of Co metal was present in the CF sample, leading to difficulty in acquiring the pre-edge and EXAFS spectra. Thus, we only used peaks of the white line, which was related to the 1 *s* to 4*p* transition. The oxidation state of Co was calculated from the white line peak position and the Co oxidation linear function using a standard reference (Supplementary Fig. 13). The XANES spectrum of CF-O is almost similar to that of raw CF, but a small broad peak is observed at 7722 eV. This suggested that a highly thin Co oxide layer was formed on the CF surface after the alkaline oxidation. The spectrum of CF-SO peaked at 7725 eV and was more intense and higher than that of CF-O. This indicates that the Co oxidation state of CF-SO is higher due to the greater number of active Co species compared to that of CF-O. This indicates that the Co oxidation state of CF-SO is higher due to the greater number of active Co species compared to that of CF-O. These morphological and electronic structural characterization results indicate that S increases the surface roughness and converts the CF electrode to a higher oxidation state under alkaline OER conditions. In general, metallic Co is converted to CoO under alkaline OER conditions, which is consistent with the results obtained for CF-O. Sulfur treatment forms electrodes with a high proportion of cobalt sulfides, including S element, which are less electronegative compared to oxygen. Under alkaline OER conditions, S is dissolved and exchanged with oxygen species (O^2‒^, OH^‒^), reconstructing the oxygen-abundant structure with high Co oxidation state. Cl ions present in Fe treatment also act like S ions, forming abundant active Co^3+^ species on the rough CF surface.

### Electrochemical properties and water splitting cell test of the fabricated CF electrode

**Fig. 3:**
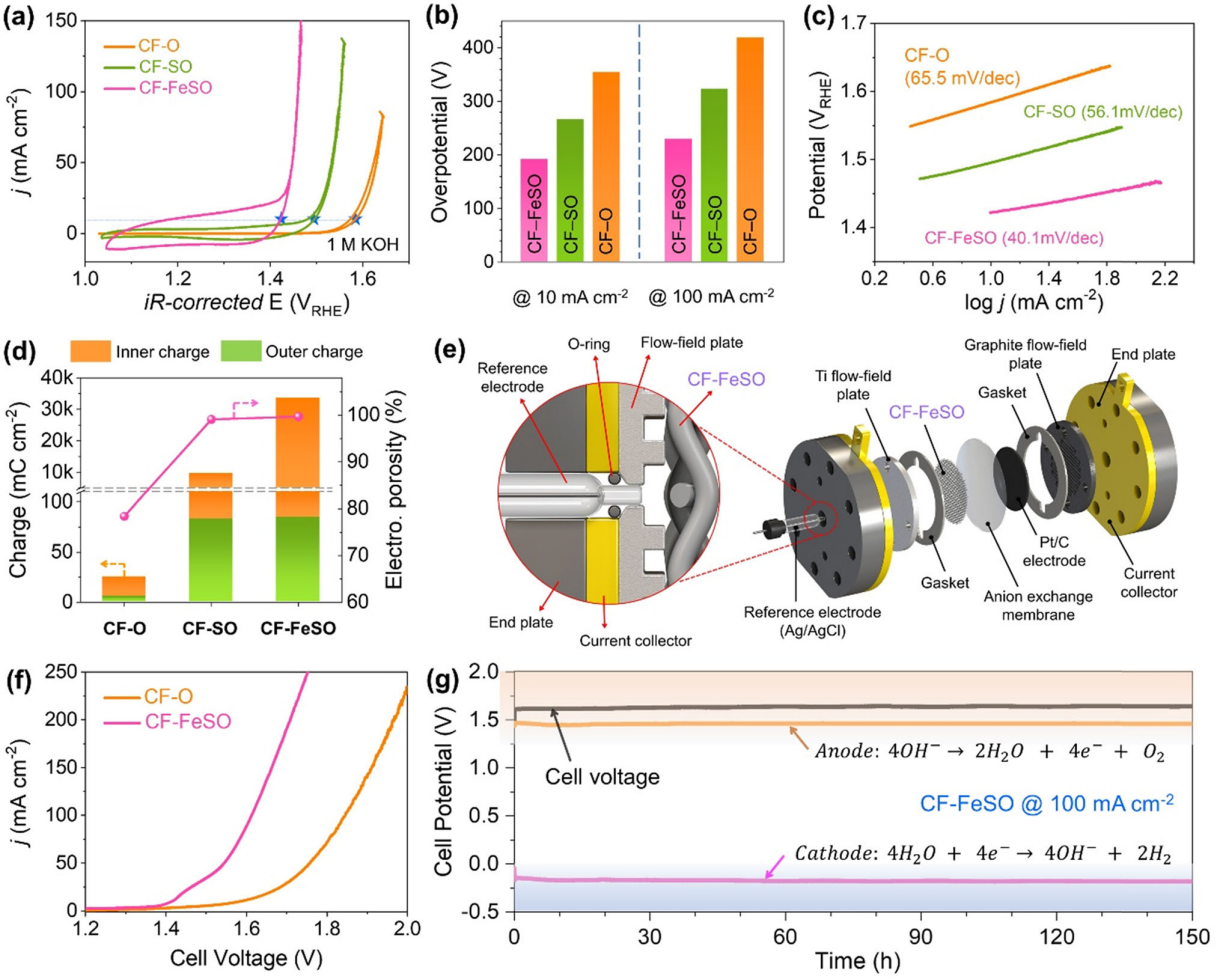
Electrochemical catalytic activity and properties of the CF-based electrodes. **a** Electrocatalytic OER activity of CF-based electrode in 1 M KOH. **b** OER activities of the catalysts expressed as overpotentials required for 10 and 100 mA cm^–2^. **c** Tafel slopes for CF-based electrode. **d** Internal and external voltammetric charge densities of the CF-based electrode. **e** Scheme of water electrolysis single cell. **f** LSV of CF-O and CF-FeSO in water electrolysis single cell. **g** Durability test in water electrolysis single cell. Cell voltage, anode potential, and cathode potential at 100 mA cm^–2^ in 1 M KOH.

We examined the OER catalytic activity of the CF-based electrodes in O_2_-saturated 1 M KOH electrolyte using a conventional three-electrode system. The cyclic voltammetry (CV) curves in Fig. 3a reveal that CF-FeSO exhibited much higher OER activity than CF-SO and CF-O. The overpotential of each electrode at 10 and 100 mA cm^–2^ is illustrated in Fig. 3b. CF-FeSO exhibits low overpotentials of 192 and 230 mV at current densities of 10 and 100 mA cm^–2^, respectively, which are considerably lower than those of CF-SO (266 and 323 mV) and CF-O (354 and 419 mV). Moreover, the Tafel slope for CF-FeSO was 40.1 mV dec^–1^, which is less than those of CF-SO (56.1 mv dec^–1^) and CF-O (65.5 mV dec^–1^) (Fig. 3c). The overpotentials and Tafel slopes indicated that Fe and S treatment of the CF enhanced its intrinsic catalytic activity for the OER. To investigate the reasons for the enhanced OER activity of CF-FeSO, we calculated the internal and external voltammetric charge densities and the electrochemical porosities by CV at different scan rates (Fig. 3c, d, Supplementary Figs. 14 and 15)^46–48^. Internal and external voltammetric charge densities illustrate theoretical charge of inside and surface, respectively. Electrochemical porosity is defined as the ratio of internal voltammetric charge to external voltammetric charge, showing roughness of electrode. The detailed calculation methods are shown in SI. The results of calculated charge density and electrochemical porosity suggested that S treatment not only significantly increased the electrode roughness, but also produced abundant catalytically active sites. These results are consistent with the CV area and XANES spectra. In the S treatment step, the S anion is incorporated into the interior of CF and then replaced by oxygen species as the sulfur dissolves in the alkaline OER conditions. This phenomenon significantly increases electrochemical porosity of CF-S. In addition, in the Fe treatment step, Cl anion play a similar role to S anion to form a highly porous surface and expand the active surface area of CF-FeSO. To separately elucidate the effect of surface area and intrinsic catalytic activity, we compared the OER activities of nanopowdered CP-O and CP-FeSO and their results are shown in Supplementary Fig. 16. In the case of the nanopowder-type, there is no significant change in the physical structure. The electrochemically active surface areas (ECSA) of CP-FeSO are slightly higher than that of CP-O, as shown in Supplementary Fig. 17. Despite a little difference of ECSA, CP-FeSO showed significantly improved OER activity than CP-O, suggesting that the reconstituted active sites of CP-FeSO had enhanced intrinsic catalytic activity for OER. Therefore, the foam type CF-FeSO electrode showed improved OER activity due to the synergistic effect of excellent intrinsic OER activity and large surface area.

We roughly controlled the Fe content of CF-FeSO by adjusting the concentration of FeCl_3_ solution (Supplementary Fig. 18). Then the OER performance according to the Fe content of the prepared electrodes was evaluated and shown in Supplementary Fig. 19. Interestingly, the CV area increased with increasing FeCl_3_ concentration because the Cl ions reconstituted the CF surface and, thus, the surface area increased. These results lead to a non-linear relationship between the Fe content of CF-FeSO and the FeCl_3_ concentration. The OER activity was similarly increased when the FeCl_3_ concentration was between 0.05 ~ 0.5 M, which means that the incorporated Fe enhances the intrinsic activity for OER, but Fe content above a certain amount no longer effects OER performance. At a FeCl_3_ concentration above 1 M, OER performance deteriorates due to corrosion of CF substrates.

To ensure stability, scalability, and feasibility at an industrial scale, single water electrolysis cell test was conducted in 1 M KOH with Pt/C electrode as a cathode electrode. Details of the water electrolysis cell with reference electrode for detecting anode and cathode potentials are described in Fig. 3f. CF-FeSO exhibited a cell voltage of 1.61 V at a current density of 100 mA cm^‒2^, which is 230 mV lower than that of CF-O (Fig. 3g). Figure 3h shows the cell voltage, anode potential, and cathode potential of the alkaline water electrolysis cell with the CF-FeSO electrode during the 150 h measurement at 100 mA cm^–2^. The cell voltage of CF-FeSO was maintained at 1.62 V with a low overpotential of 200 mV for the OER, indicating remarkable stability of CF-FeSO. As shown in Supplementary Fig. 20, the SEM and EDS results confirmed that the structure and composition of CF-FeSO were also well maintained after the durability tests. This confirmed the stability and activity of the CF-FeSO electrode in the large-scale water electrolysis.

### In-situ/operando study for observing phase and spin state of the fabricated CF electrode

**Fig. 4:**
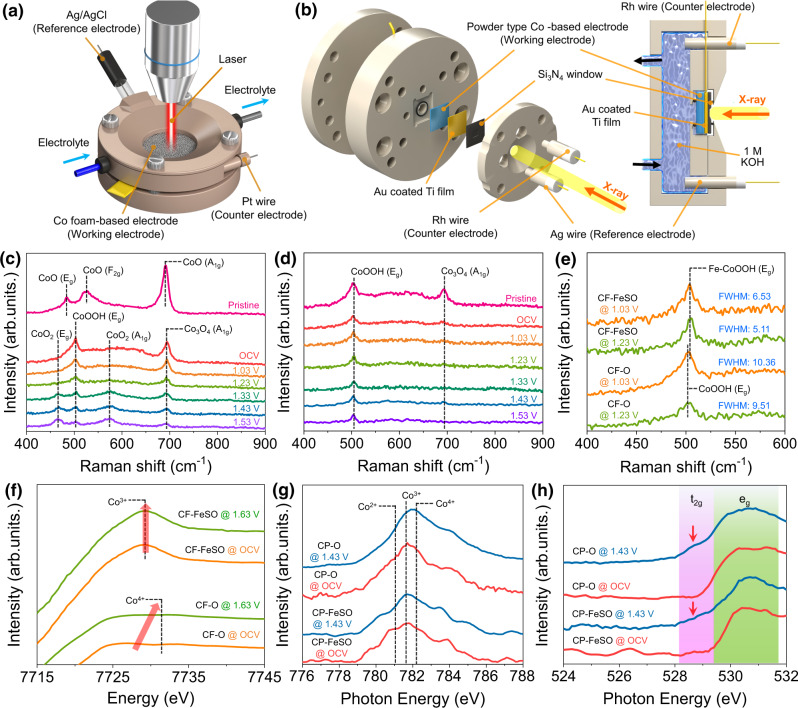
In-situ/operando spectroscopy for investigating the origin of the high OER activity of the CF-FeSO electrode. Schematic illustration of the in-situ*/operando* (**a**) Raman spectroscopy setting and **b** NEXAFS setup. **c** In-situ*/operando* Raman spectrum of the CF-O electrode. **d** In-situ*/operando* Raman spectrum of the CF-FeSO electrode. **e** In-situ*/operando* Raman spectrum of the CF-based electrode for comparing the CoOOH peak. **f** In-situ*/operando* Co K-edge XANES spectra of the CF-O and CF-FeSO electrodes. **g** In-situ*/operando* Co L-edge NEXAFS spectra of the CP-O and CP-FeSO catalysts. **h** In-situ*/operando* O K-edge NEXAFS spectra of the CP-O and CP-FeSO catalyst.

To identify the actual OER active site of the CF-based electrode, we employed in-situ*/operando* techniques such as Raman, XANES, and NEXAFS spectroscopies using a customized electrochemical cell (Fig. 4a, b). The phase of cobalt materials can be transformed by varying the potential and pH, suggesting that the electronic structure of cobalt materials under OER conditions will change with respect to that at the initial state. Thus, in-situ*/operando* techniques are necessary for identifying the actual active site of the Co-based catalyst. For in-situ*/operando* Raman spectral measurements, the laser has to be focused on the thin electrolyte-coated CF-based electrode to identify the phase of the electrode surface during the OER. The Raman spectrum of CF-O (Fig. 4c) exhibits the CoO phase. The CoO (E_g_ and F_2g_) peaks are transformed into the CoOOH (E_g_) and Co_3_O_4_ (A_1g_) peaks when CF-O is dipped in 1 M KOH solution under OCV condition. When the potential is increased to 1.53 V, intensities of the CoOOH (E_g_) and Co_3_O_4_ (A_1g_) peaks decrease and that of the CoO_x_ (predicted to be CoO_2_) peak is increases^28^. This trend was also observed in repeated tests, demonstrating the reversibility of transformation (Supplementary Fig. 21). The transformation CF-O (Co(II)O → Co(III)OOH + Co(II, III)_3_O_4_ → Co(VI)O_2_) due to increased potential in the alkaline solution is largely consistent with the thermodynamic theory discussed in the introduction. These results indicate that CoO_2_ is the actual active site of CF-O for the OER. On the contrary, the CoO_2_ species could not be detected in the operando Raman spectrum of CF-FeSO (Fig. 4b). Under OCV conditions, the Co_3_O_4_ (A_1g_) peak disappeared, and only the CoOOH (E_g_) peak was detected. It suggests that CoOOH is a stable phase of CF-FeSO in the alkaline solution. Even if the applied potential for the case of CF-FeSO increases to 1.53 V, only the CoOOH peak is detected, demonstrating that Fe-CoOOH is the main active site for the OER. Several Fe-CoOOH and Co_3_O_4_ species formed upon Fe and S treatment were easily converted into the single Fe-doped CoOOH phase, which was retained, and which act as an OER active site under the OER conditions.

To understand the effect of S, Cl, and Fe element in reconstruction process, CF-ClSO was manufactured by using HCl instead of FeCl_3_ and in-situ*/operando* Raman spectrum of CF-SO and CF-ClSO was acquired (Supplementary Figs. 22 and 23). In the absence of a CoO_x_ peak, the main phase of CF-SO was determined to be CoOOH and Co_3_O_4_. S treatment results in a high oxidation state of cobalt and an oxygen-abundant structure for the reconstructed CF electrode. These properties confer enhanced structural stability and inhibit the conversion to CoO_2_ species. The single CoOOH phase of CF-ClSO suggests that the role of Cl is promoting the conversion to the CoOOH phase by accelerating reconstruction and the role of Fe is only modulating electronic structure of Co for OER. The OER performance of CF-ClSO clearly reveals effects of Cl and Fe element (Supplementary Fig. 23). To verify the peaks in the Raman spectrum, isotopic substitution using D_2_O instead of H_2_O was conducted. The observed D_2_O peak confirmed the in-situ condition and isotropic substitution experiment (Supplementary Fig. 24). The CoOOH (E_g_) peak was blue-shifted, suggesting the replacement of hydrogen with deuterium (Supplementary Figs. 25 and 26). In contrast, the CoO_x_ peaks showed no shift in the isotopic substitution experiment, suggesting that there was no contribution from hydrogen in the CoO_2_ peak, which is consistent with a previous report^29^. The CoOOH peaks of CF-FeSO were red-shifted with respect to those of CF-O because of the larger radius of Fe^3+^ than Co^3+^ (Fig. 4e). This confirmed the Fe-CoOOH phase of CF-FeSO under the OER conditions. The OER intermediates were also observed by in-situ*/operando* Raman spectroscopy (Supplementary Fig. 27). Peaks corresponding to the superoxo species (OO^–^) were observed for the CF-O electrode; however, CF-FeSO showed no peaks for the OER intermediates. This suggests that the rate determining step (RDS) in the OER of CoO_2_ was the release of O_2_^28^ whereas, RDS of Fe-CoOOH would be the deprotonation step. The oxidation state of the Co-based electrode measured by in-situ*/operando* XAFS can further confirm the transition of the Co phase under OER conditions. As shown in Fig. 4f and Supplementary Figs. 28–30, the change in the XANES spectrum is small due to the metallic Co substrates. The small and broad XANES peak of CF-O shifted to 7725 eV at OCV, suggesting that the surface was oxidized in alkaline solution. When the applied potential increases to 1.63 V, the peak shifts to a higher energy, indicating a phase transition of Co at the surface. In contrast, CF-FeSO showed stable oxidation states of Co^3+^ and Fe^3+^ from OCV up to 1.63 V (Supplementary Fig. 31), confirming that the Fe-doped CoOOH sites maintained stable phases under harsh OER condition. These trends are also observed in nanopowder-type samples (Supplementary Fig. 32), so the results of XANES support the observations of in-situ*/operando* Raman spectroscopy.

To closely observe the spin state and oxidation states at catalyst surface under OER conditions, we conducted in-situ*/operando* NEXAFS spectroscopy. The foam type electrode is considerably difficult to adjust in the in-situ*/operando* NEXAFS cell owing to the harsh surface. We prepared CP-FeSO powder using a method similar to the synthesis method of CF-FeSO and compared it with CP-O. The catalyst was loaded on 20 nm Au, and 10 nm Ti was coated on 100 nm SiN window. The catalyst-coated SiN window was attached to a customized NEXAFS cell (Fig. 4b). Under OCV conditions, CP-O exhibited Co^2+^ and Co^3+^ peaks with filled *t*_*2g*_ orbitals, indicating that CP-O contained Co^2+^ and Co^3+^ (LS) species (Fig. 4g, h). When the potential increased to 1.43 V, the Co L-edge peak of CP-O was shifted to higher energy, with a clear appearance of the *t*_*2g*_ peak (O K-edge spectrum) of CoO. The shifted broad Co L-edge peak indicates a mixture of Co^2+^, Co^3+^, and some converted Co^4+^(CoO_2_) species. These results agreed with the in-situ*/operando* Raman spectrum. The *t*_*2g*_ peak of CP-O suggests that the CoO_2_ generated during the OER would have a HS (*t*_*2g*_^3^*e*_*g*_^2^) state. CP-FeSO also exhibited a higher spin state under OER conditions, but the trend was slightly different. At 1.43 V, the Co L-edge of CP-FeSO almost maintained the position of the main peaks with decreasing Co^2+^ species, indicating a high proportion of the Co^3+^ state (Fe-CoOOH) of the CP-FeSO catalyst under OER conditions. Despite the high proportion of Co^3+^ species, a small t_2g_ peak of CP-FeSO was detected. This suggested that Fe-CoOOH in CP-FeSO transformed to an intermediate spin (IS, *t*_*2g*_^5^*e*_*g*_^1^) state from the LS state during the OER. All the cobalt catalysts were converted to the high spin state under OER conditions. The increased intensity of the *t*_*2g*_ peak of the O K-edge NEXAFS was also observed in in-situ*/operando* NEXAFS study of an iridium catalyst under acidic conditions^49^. Pfeifer et al. reported that amorphous iridium oxide possesses electrophilic oxygen under OER conditions. We anticipated that a similar phenomenon occurs in the Co-based catalyst under alkaline OER conditions. The oxygen species of the Co-based catalyst are expected to have a higher electrophilic character during the OER and are converted to weaker field ligands, thus resulting in the higher spin state of the cobalt catalyst under OER conditions.

### Origin of the high OER performance of CF-FeSO

**Fig. 5:**
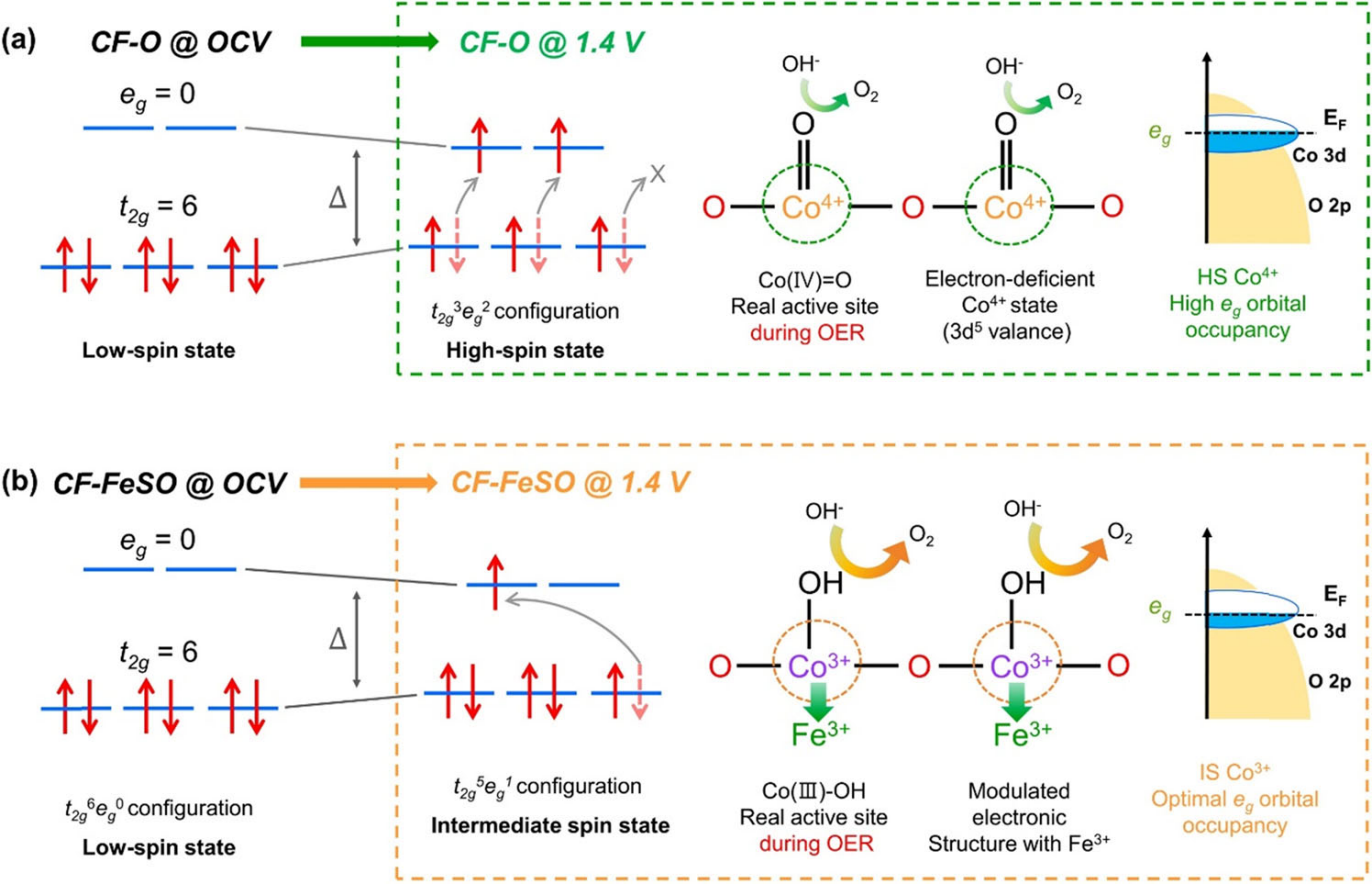
Schematic illustration of the proposed origin of the high OER activity for CF-FeSO electrode. Spin state under OCV and OER conditions and chemical phase and Co 3d– O 2p overlap under OER conditions for **a** CF-O and **b** CF-FeSO. Transparent dotted arrows indicate the state before the OER and red arrows represent the converted spin state during the OER.

The OER performance of the CF-based electrode is highly affected by he properties of the active sites under OER conditions. The estimated real active sites and their estimated electronic configurations during the OER are shown in Fig. 5. The CoO_2_ phase in CF-O, for which the active site is Co^4+^ under OER, exhibited 3d^5^ valence electronic configuration with a HS state. Shao-Horn *et al*. proposed that the optimal *e*_*g*_-orbital occupancy for the binding of OER intermediates is almost 1.2^37^. The HS CoO_2_ phase has a half-filled *e*_*g*_ orbital, with an occupancy of ≈ 2. This is higher than the optimal value and renders the electrophilic oxygen lattice as a weaker field ligand. Furthermore, the CoO_2_ phase with half-filled *e*_*g*_ orbital has weak bonding with the adsorbed oxygen intermediates, thereby enhancing their electrophilicity^50^. These electronic structures of the HS CoO_2_ could stabilize the electrophilic superoxo intermediates (OO^–^) to a higher extent, as observed by Raman spectroscopy (Supplementary Fig. 27). Owing to this, the slow dioxygen release becomes the RDS, thus explaining the low OER activity of CF-O. On the other hand, the CF-FeSO electrode possessed abundant Fe-CoOOH species under real OER conditions without changing to the CoO_2_ species. Magnussen *et al*. analyzed the stable CoOOH structure by *operando* surface X-ray diffraction^25^. Defects and edge sites of the Co materials are expected to initiate the phase transition. The CoOOH (100) film possesses the ideal three-fold coordinated µ_3_-O site with very low defects (µ_2_-O site), resulting in a stable phase in the OER reaction. In the in operando Raman spectrum, the CoOOH peak of CF-FeSO was sharper than that of CF-O, suggesting that the reconstructed Fe-CoOOH in alkaline media has lower defects than the phase-transferred CoOOH (Fig. 4d)^21^. This result means that under OER conditions, a stable Fe-CoOOH phase of CF-FeSO is produced, which is equivalent to the elaborately prepared CoOOH^25^. Furthermore, the Fe-CoOOH phase of CF-FeSO changed to an IS state (*t*_*2g*_^5^*e*_*g*_^1^) from the LS state (*t*_*2g*_^6^*e*_*g*_^0^) under OER conditions, as observed by in-situ*/operando* NEXAFS spectroscopy. Recent studies suggest that the Fe-substituted IS state of Co^3+^ leads to optimal filling of the e_g_ orbital and enhanced covalency, thus promoting the OER catalytic activity^33,34^. The IS state of CoOOH (Co^3+^) showed 1 of e_g_-orbital occupancy, possess slightly strong adsorption energy for the OER. The HS state of Fe^3+^ exhibited the 3d^5^ (*t*_*2g*_^3^*e*_*g*_^2^) configuration, which is expected to have more filled e_g_ orbitals and weaker bonding with the adsorbed oxygen intermediates than IS state of CoOOH. We expected that the local configuration of Fe^3+^ and CoOOH could lead to a modulated electronic structure with optimal adsorption energy of OER intermediates, leading to high OER catalytic activity^51^. The RDS of Fe-CoOOH is deprotonation step which suggests thermodynamically favorable chemical step for OER^52^, revealing this optimal electronic structure. Therefore, the CF-FeSO electrode possesses many stable Fe-CoOOH phases with IS states during the OER owing to reconstruction in alkaline solution; These phases are expected to have the proper electronic structure to provide optimal adsorption energy to the OER intermediates, resulting in high OER performance. These results show that the phase and spin state of the real active sites significantly influence the catalytic activity. Furthermore, no elaborate engineering of the pristine catalyst is required. This facile electrochemical reconstruction for fabricating electrode is an effective strategy at an industrial scale for improving the OER performance.

## Discussion

In this work, we demonstrate a facile and efficient strategy to fabricate a highly active and scalable electrode for the OER, without requiring detailed engineering of the catalyst. The CF-FeSO electrode was manufactured by iron dipping, sulfur heat treatment, and reconstruction under OER conditions. CF-FeSO exhibited remarkable OER activity (192 mV at 10 mA cm^‒2^ and 230 mV at 100 mA cm^‒2^) and durability (150 h at 100 mA cm^‒2^) not only for a three-electrode system but also for a large-scale water electrolysis cell. CF-FeSO has a highly porous and rough surface, leading to an enlarged active surface area. Under alkaline conditions, the active site of CF-FeSO is reconstructed to the Fe-CoOOH species. In-situ*/operando* Raman spectroscopy and XAFS spectroscopy show that the Fe-CoOOH species, known to be an intrinsically highly active species for the OER, was retained under the OER conditions and did not convert to the CoO_2_ species, which have low activity. Based on the results of in-situ*/operando* NEXAFS spectroscopy, the spin state of the Co-based catalyst increased under the OER operating conditions. Especially, Fe-CoOOH was converted to the IS state from LS state, resulting in optimal electronic structure and energy of the OER intermediates. This Fe-CoOOH phase and IS spin state of CF-FeSO under the reaction conditions lead to superior OER performance. Our work emphasizes the importance of real active sites during the OER and provides a new viewpoint for the fabrication of electrode by means of electrochemical reconstruction.

## Methods

### Materials

KOH (90% flake), sulfur, deuterium oxide (D_2_O), and FeCl_3_ were purchased from Sigma Aldrich. CF was bought from alantum. The DI water used in this work was prepared using an arium mini lab water system (Sartorius). The anion exchange membrane (AEM, Sustainion® X37-50 grade T) for the single cell test was purchased from Dioxide Materials. All products were used as received without further purification.

### Preparation of Co foam-based electrodes

A piece of CF (1 × 2 cm^2^) was washed with DI water and dried under N_2_. For the iron treatment, 0.25 M FeCl_3_ solution was deposited on the porous CF by a dip coating process and then dried using a convection oven at 70 °C (denoted as CF-Fe). The prepared CF-Fe electrode was sulfurized by heat treatment with sulfur. Sulfur powder (200 mg) and Fe-CF were put into each side of a quartz boat. The quartz boat with sulfur powder and Fe-CF was put into the center of a furnace tube. The side of the quartz boat with CF was placed downstream of the furnace. The furnace temperature was raised to 300 °C (at 10 °C/min) and maintained for 5 min. The furnace was cooled down to room temperature. The electrochemical reconstruction process was conducted in a three-electrode system with iron and sulfur-treated CF (denoted as CF-FeS) as the working electrode, graphite electrode as the counter electrode, and Hg/HgO as the reference electrode. For electrochemical reconstruction process, a current density of 100 mA cm^‒2^ was applied for 10 min. To check the completion of electrochemical reconstruction, cyclic voltammetry was performed in the potential range 1–1.8 V vs RHE at 100 mV s^‒1^ for 50 cycles, 10 mV s^‒1^ for 10 cycles, and 2 mV s^‒1^ for 2 cycles. If the electrochemical reconstruction process is not completed, the CV will appear slanted. After the electrochemical reconstruction, the iron and sulfur-treated CF (CF-FeSO) was washed with DI water. The preparation process of CF-SO was same as that of CF-FeSO, except that there was no iron treatment process. The preparation process of CF-O was same as that of CF-FeSO, except that there were no iron and sulfur treatment processes. The preparation process of CF-ClSO was same as that of CF-FeSO, except that 0.75 M HCl was used instead of 0.25 M FeCl_3_. The preparation process of powder-type CP-FeSO was same as that of CF-FeSO, except for the starting material—commercial CoO powder (Sigma Aldrich) was used as the starting material.

### Electrochemical measurements

The electrochemical tests were performed on potentiostat VSP (biologic) using a Hg/HgO electrode as the reference electrode and graphite electrode as the counter electrode. An O_2_-saturated 1 M KOH solution was used as the electrolyte. The measured potential was calibrated to the RHE. The Hg/HgO reference electrode was calibrated using CV under hydrogen-saturated conditions. After electrochemical reconstruction, the electrolyte was replaced with a new electrolyte owing to sulfur oxidation. The catalytic activity of the fabricated electrode for the OER was measured from the CV curves at a scan rate of 2 mV s^‒1^. Ohmic resistance was measured using electrochemical impedance spectroscopy from 1000 to 0.1 Hz to compensate the iR loss. The alkaline water electrolyzer test was conducted using a 10 cm^2^ single cell with two symmetric electrodes as both cathode and anode. To prepare the cathode, commercial Pt/C (Tanaka Kikinzoku Kogyo 46%) was sprayed on carbon paper (Sigracet SGL 39BB). The Pt loading was 0.4 mg cm^‒2^. The sustainion anion exchange membrane (PTFE Supported Sustainion® 37-50, Dioxide Materials) was used to separate both the electrodes. KOH solution (1 M) was allowed to flow to the anode and cathode sides using a peristaltic pump. The Ag/AgCl (3 M NaCl) reference was adapted to the anode-side for measuring the cathode and anode potentials. The cathode and anode potentials were calculated using the following equation: Cell voltage = anode potential – cathode potential – *IR*, where *I* and *R* are the measured current and ohmic resistance, respectively.

### Material characterization

For characterization of the electrode, Raman spectroscopy (Renishaw) was conducted at room temperature using a 785 nm laser for identifying the phase of the cobalt-based electrode. X-ray photoelectron spectroscopy (XPS, ULVAC PHI, VersaProbe PHI 5000) was performed to investigate the chemical state of the cobalt-based electrode. X-ray absorption spectroscopy (XAS) and near edge XAS (NEXAFS) spectroscopy were conducted for analyzing the chemical state of the catalyst at the 1D and 10D beamline of the Pohang Accelerator Laboratory (PAL), Pohang, South Korea. Morphology and energy dispersive spectroscopy (EDS) of the Co foam-based electrode were analyzed by SEM (Hitachi, Regulus 8230). The morphologies of the fabricated electrode and catalysts were measured by high-resolution transmission electron microscopy (HR-TEM, FEI Talos F200X). In-situ*/operando* analysis techniques are described in the Supplementary Information.

## Supplementary information

Supplementary Information

## Data Availability

Source data are provided with this paper.

## Source data

Source Data
