# Supplementary material for: Electrode reconstruction strategy for oxygen evolution reaction: maintaining Fe-CoOOH phase with intermediate-spin state during electrolysis
Source: Nat Commun. 2022 Feb 1;13:605. doi: 10.1038/s41467-022-28260-5 (PMC8807628; doi:10.1038/s41467-022-28260-5)
Supplement: Supplementary file 1 — Supplementary Information [file 41467_2022_28260_MOESM1_ESM.pdf]

Supplementary Information

Electrode reconstruction strategy for oxygen evolution reaction: maintaining Fe-CoOOH phase with intermediate-spin state during electrolysis

Woong Hee Lee [1, 2], Man Ho Han [1, 3], Young-Jin Ko [1], Byoung Koun Min [1, 4],
Keun Hwa Chae [5] *, Hyung-Suk Oh [1, 6, 7] *

[1] *Clean Energy Research Center, Korea Institute of Science and Technology (KIST), Hwarang-ro 14-gil 5, Seongbuk-gu, Seoul 02792, Republic of Korea*

[2] *Department of Chemistry, Seoul National University, Seoul 08826, Republic of Korea*

[3] *Department of Chemical and Biological Engineering, Korea University, Anamdong-5-Ga, Seoul 02841, Republic of Korea*

[4] *Graduate School of Energy and Environment (KU-KIST Green School), Korea University, 145 Anam-ro, Seongbuk-gu, Seoul 02841, Republic of Korea*

[5] *Advanced Analysis Center, Korea Institute of Science and Technology (KIST), Hwarang-ro 14-gil 5, Seongbuk-gu, Seoul 02792, Republic of Korea*

[6] *Division of Energy and Environmental Technology, KIST school, Korea University of Science and Technology, Seoul 02792, Republic of Korea*

[7] *KHU-KIST Department of Conversing Science and Technology, Kyung Hee University, Seoul 02447, Republic of Korea*

***Corresponding Authors**
E-mail: khchae@kist.re.kr (K. H. Chae), hyung-suk.oh@kist.re.kr (H. -S. Oh)
Tel.: +82 (0)2 958 5292

Contents **Page**

○ Supplementary Figures 1 ~ 20 .... S3

○ Supplementary Figures 21 ~ 27 .... S23

> *In-situ/operando* Raman spectrum results

○ Supplementary Figures 28 ~ 32 .... S30

> *In-situ/operando* X-ray absorption spectroscopy (XAS)

○ Supplementary Note 1 .... S35

○ Supplementary Note 2 .... S36

○ References S37

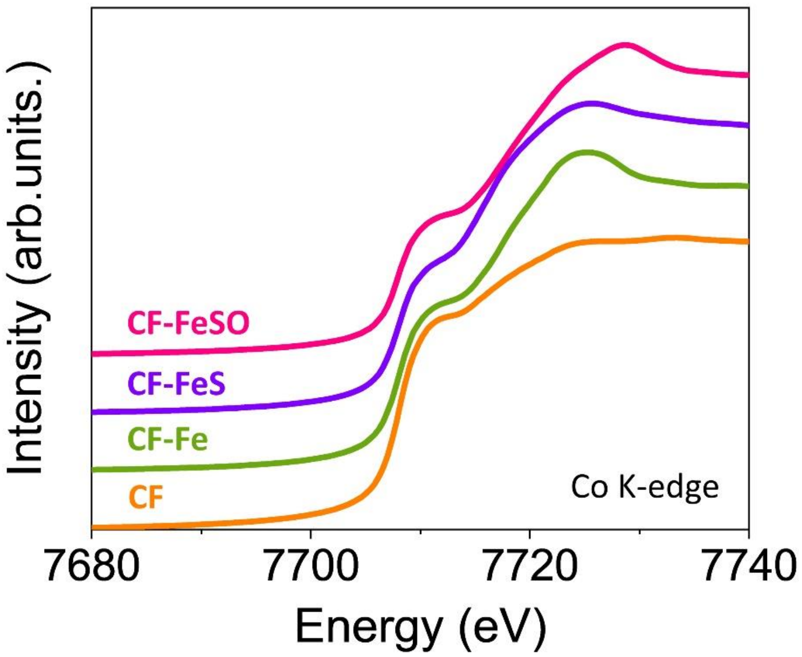

Supplementary Figure 1. **Comparison of Co K-edge XANES spectra.** X-ray absorption spectroscopy (XAS) results of Co-based electrodes in each synthesis step to prepare CF-FeSO (CF, CF-Fe, and CF-FeS). Foam-type electrode samples are processed in *ex-situ* conditions. XAS was conducted at the 1D beamline of the Pohang Accelerator Laboratory (PAL), Pohang, South Korea.

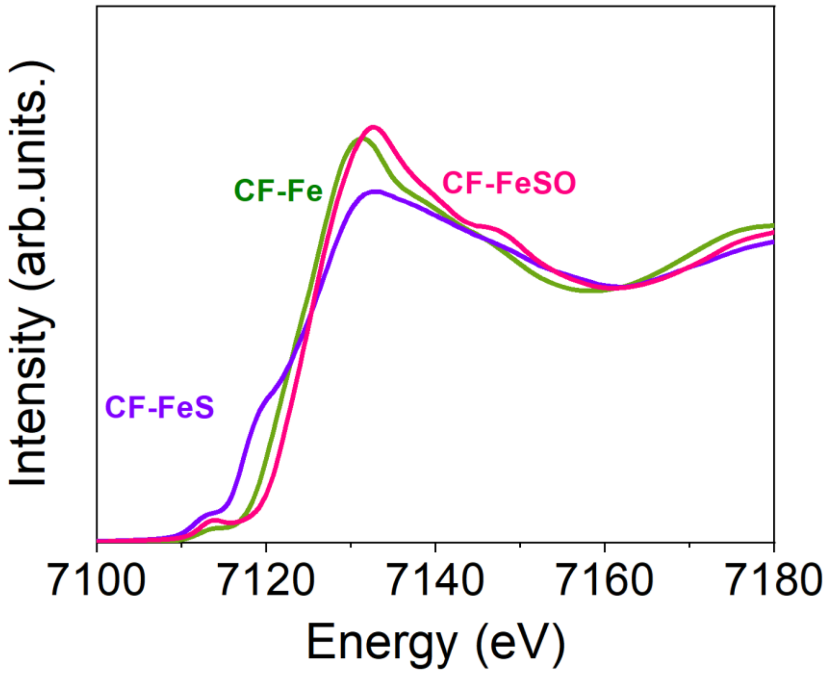

Supplementary Figure 2. Comparison of Fe K-edge XANES spectra. X-ray absorption spectroscopy (XAS) results of Co-based electrodes in each synthesis step to prepare CF-FeSO (CF-Fe, and CF-FeS). Foam-type electrode samples are processed in *ex-situ* conditions. XAS was conducted at the 1D beamline of the Pohang Accelerator Laboratory (PAL), Pohang, South Korea.

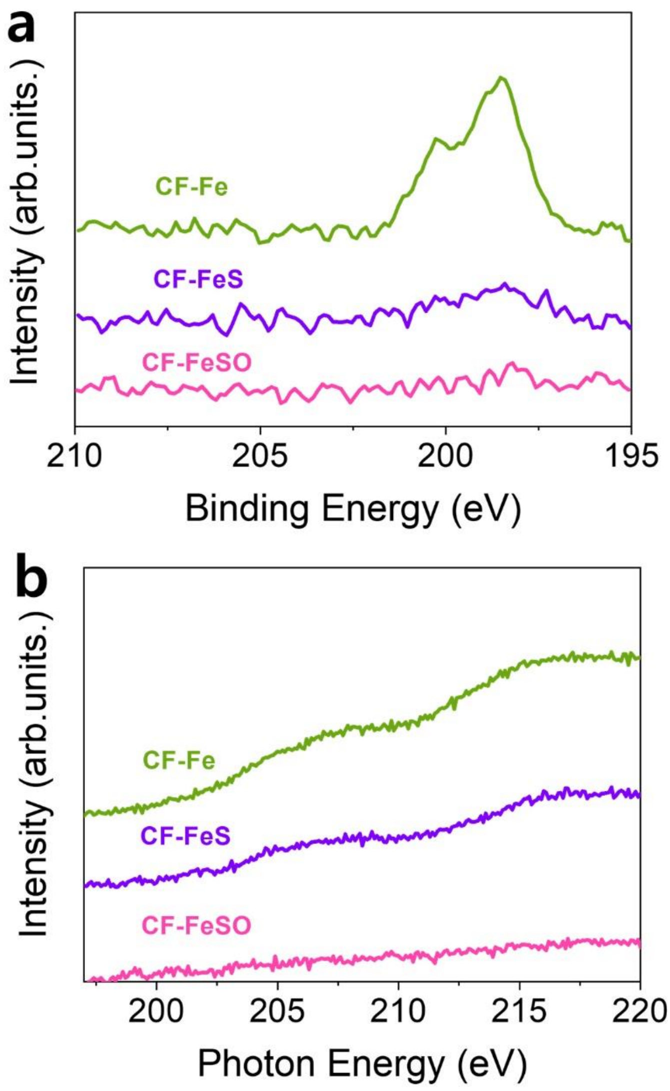

Supplementary Figure 3. XPS and NEXAFS spectrum in the manufacturing process stage of CF-FeSO electrode. (a) Cl 2p XPS and (b) Cl K-edge NEXAFS spectrum of each electrode in the manufacturing process stage of CF-FeSO electrode.

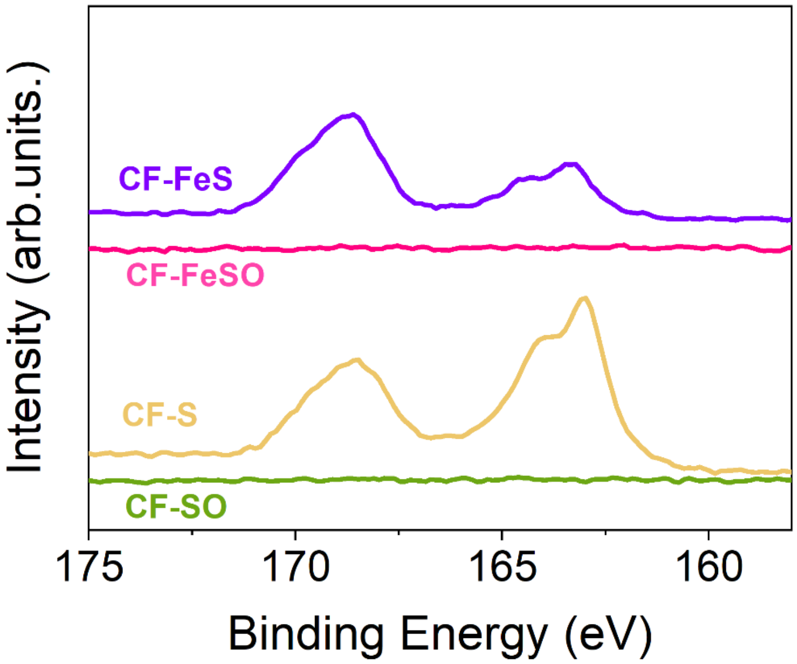

Supplementary Figure 4. **Comparison of S 2p XPS spectra of CF-based electrodes.** XPS results to understand the effect of electrochemical treatment on the sulfur of the electrode before and after electrochemical oxidation.

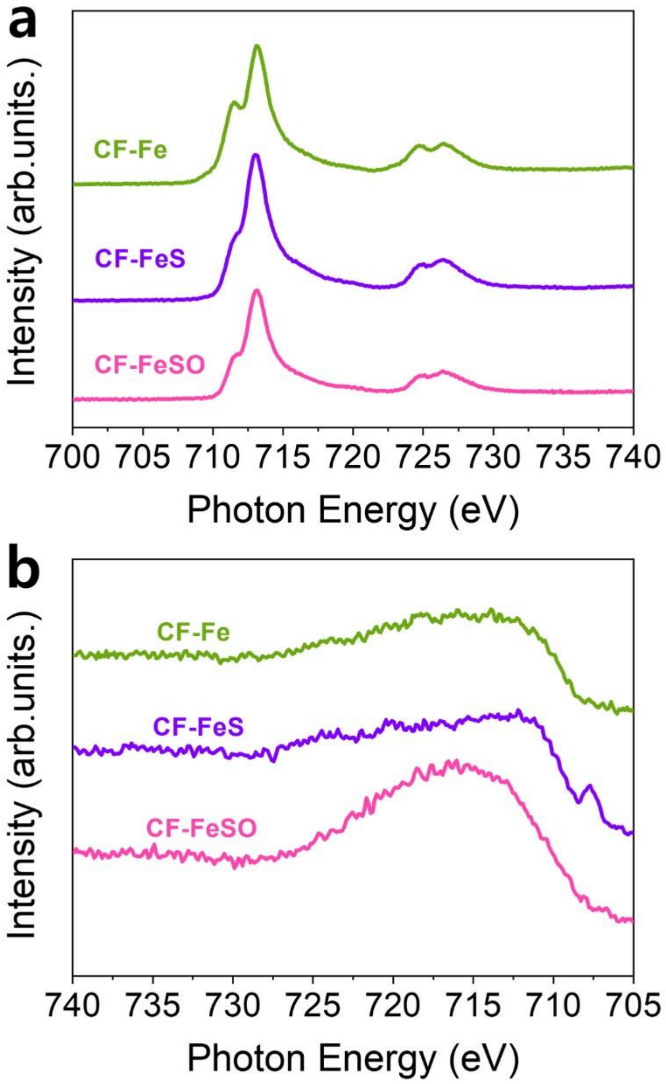

Supplementary Figure 5. **NEXAFS and XPS spectrum in the manufacturing process stage of CF-FeSO electrode**. (a) Fe L-edge NEXAFS and (b) Fe 2p XPS spectrum of each electrode in the manufacturing process stage of CF-FeSO electrode.

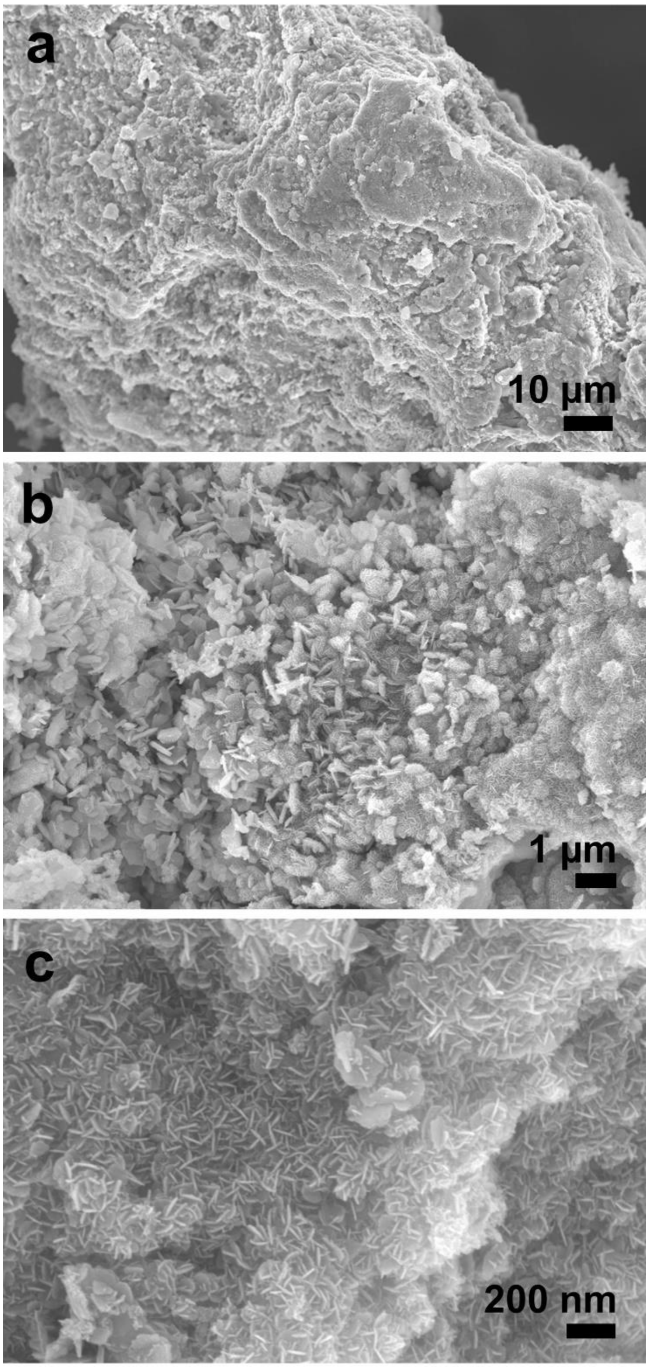

Supplementary Figure 6. SEM images of CF-FeSO electrode. (a) low, (b) medium and (c) high magnification SEM images.

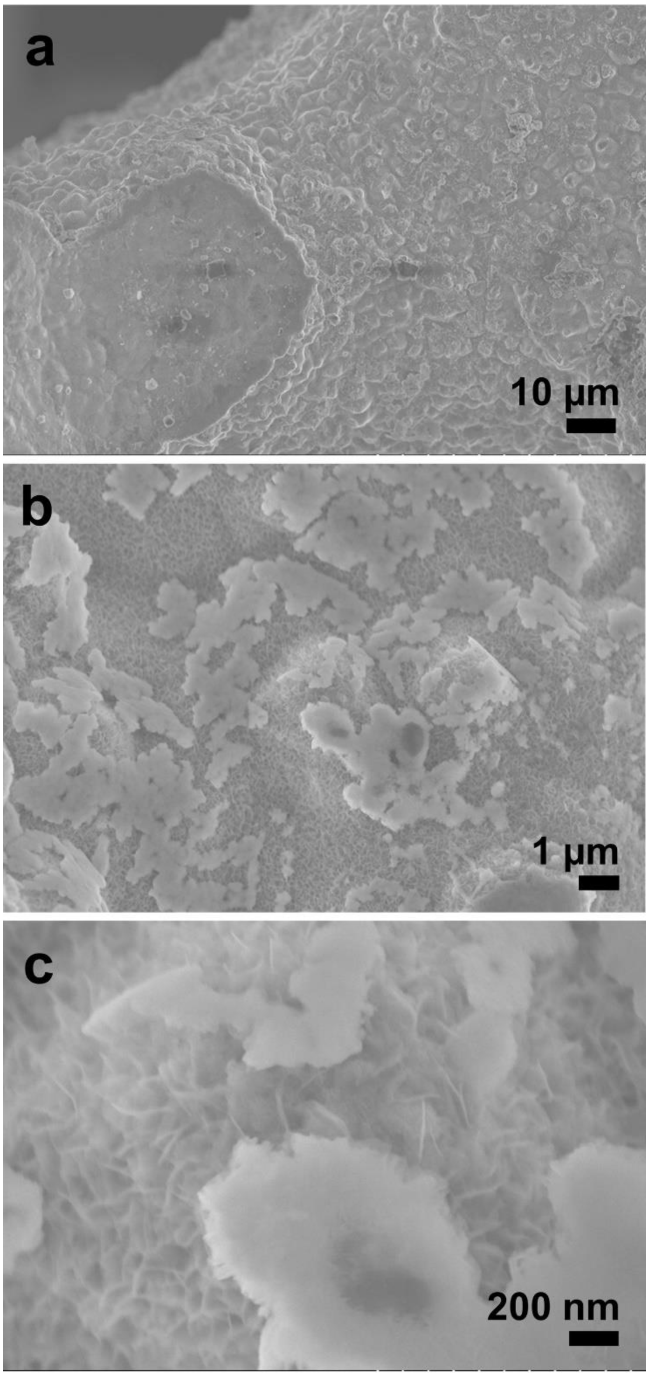

Supplementary Figure 7. SEM images of CF-O electrode. (a) low, (b) medium and (c) high magnification SEM images.

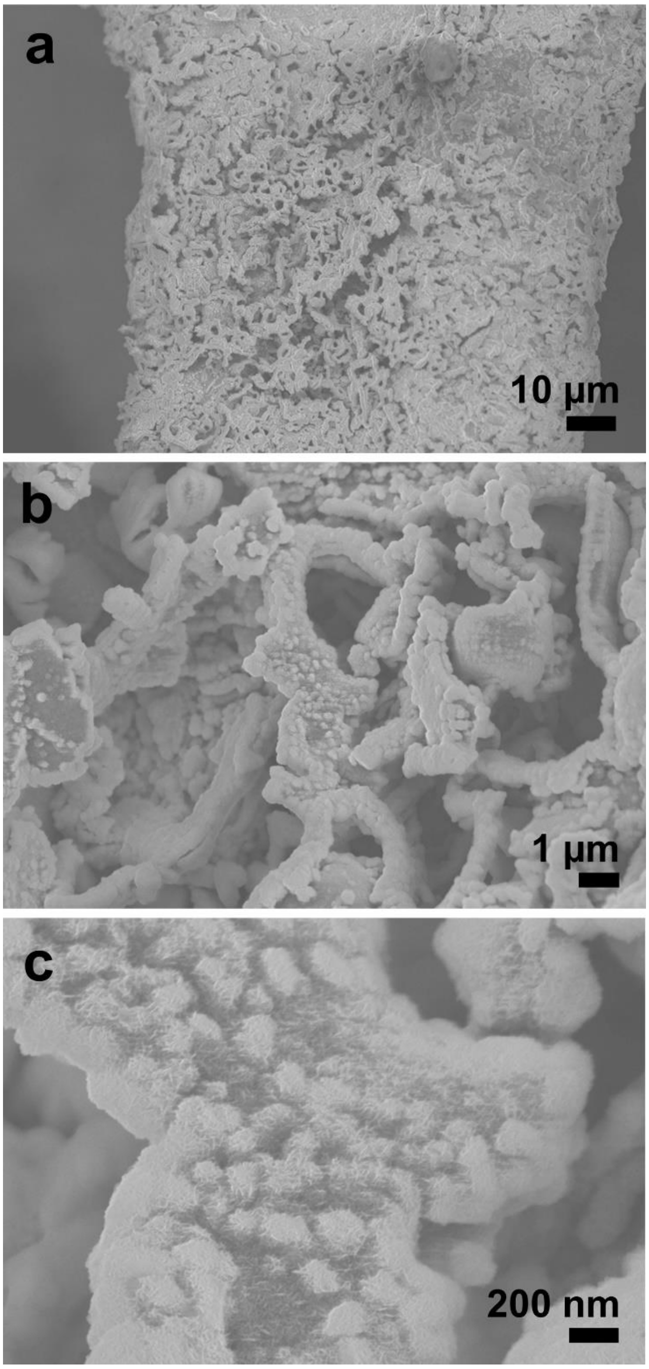

Supplementary Figure 8. SEM images of CF-SO electrode. (a) low, (b) medium and (c) high magnification SEM images.

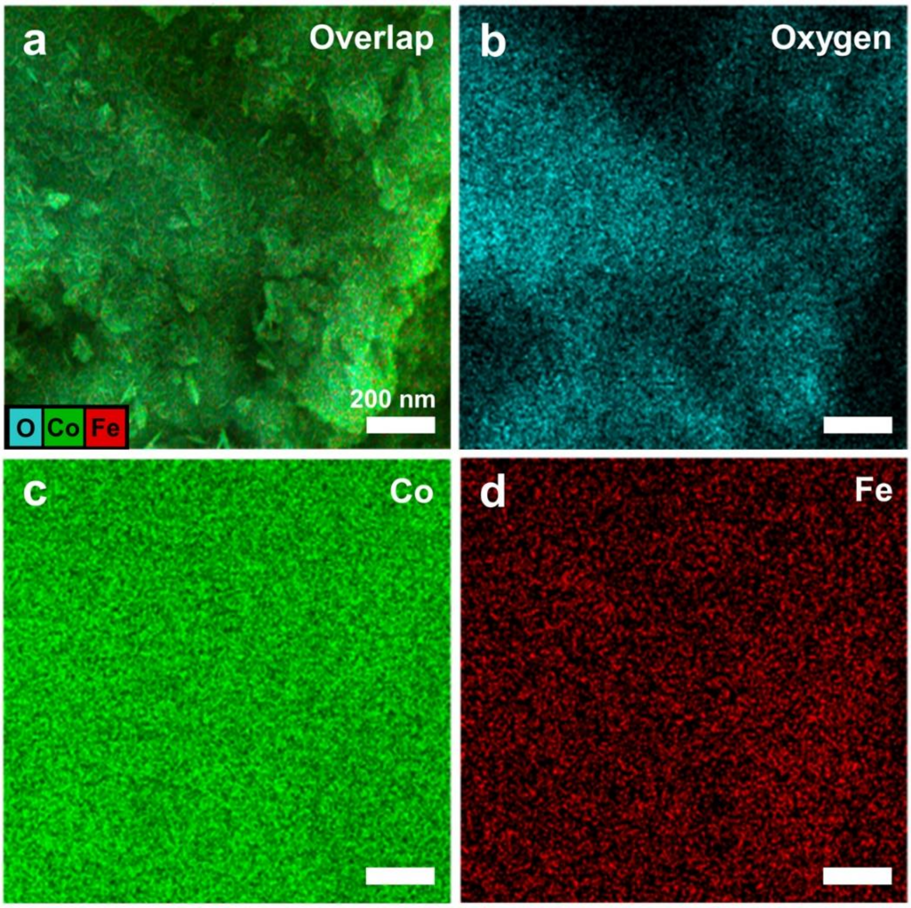

Supplementary Figure 9. **Energy-dispersive X-ray spectroscopy (EDS) elemental maps using SEM of CF-FeSO**. (a) Overlap, (b) oxygen, (c) cobalt, and (d) iron elemental mapping images.

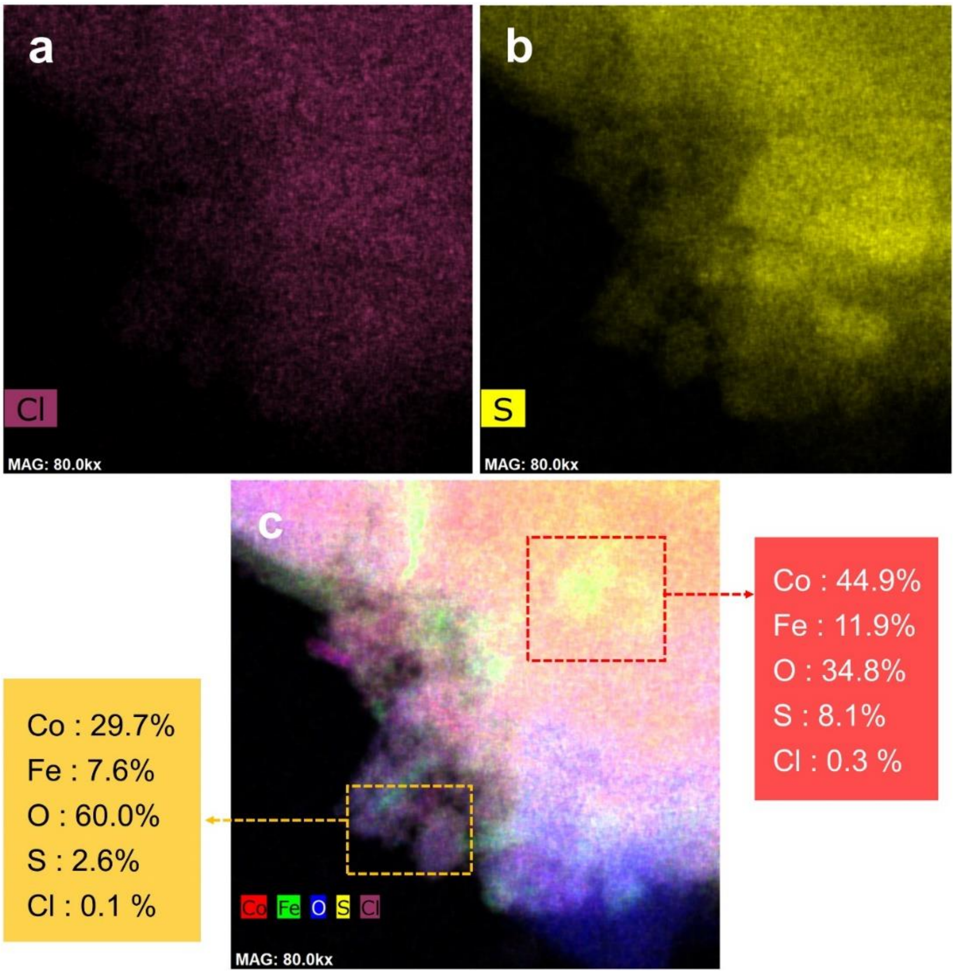

Supplementary Figure 10. Energy-dispersive X-ray spectroscopy (EDS) elemental mapping images of CF-FeSO using TEM. (a) Cl element, (b) S element, and (c) elements comparison of inside and surface of CF-FeSO.

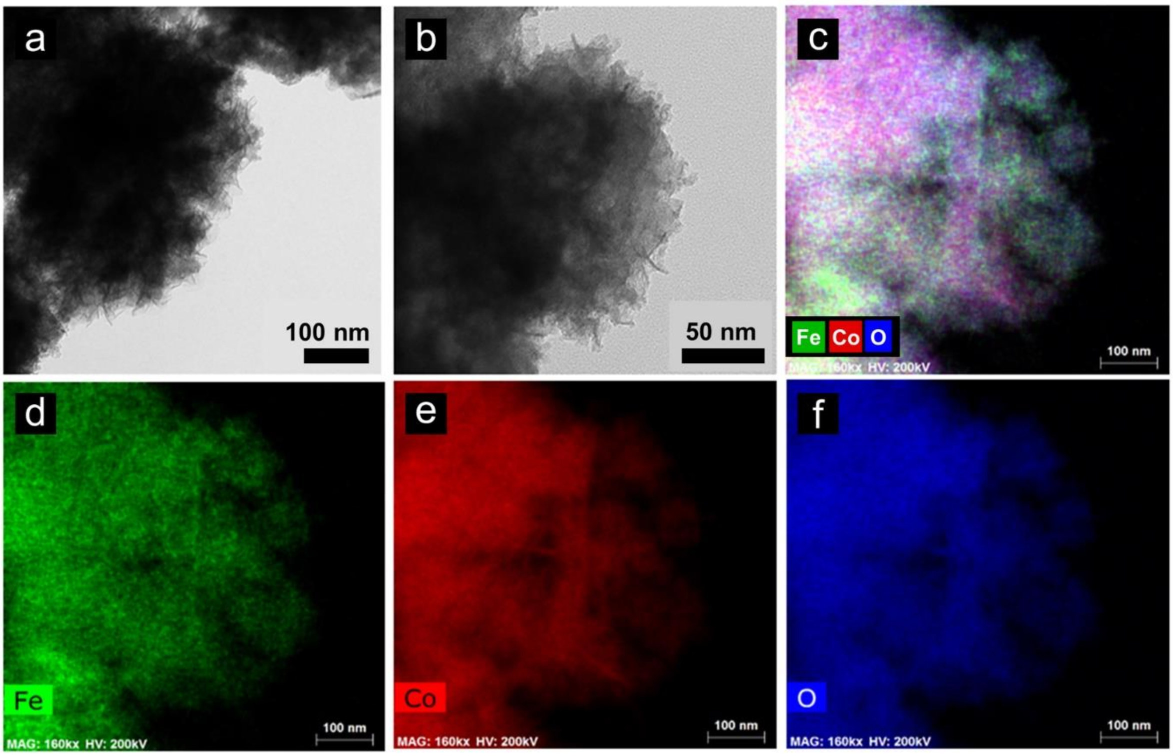

Supplementary Figure 11. HR-TEM images and EDS of powder-type CP-FeSO. (a, b) HR-TEM images of powder-type CP-FeSO. (c-f) EDS elemental maps using TEM of CP-FeSO.

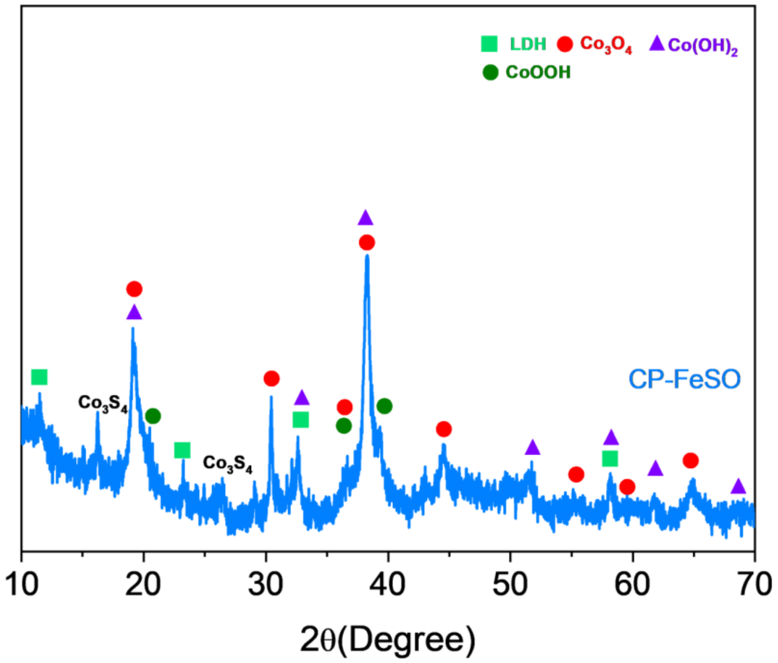

Supplementary Figure 12. XRD pattern of powder-type CP-FeSO. The measured XRD pattern indicates mixed phases (■: Layered double hydroxide (LDH), ●: Co_3O_4, ▲: $Co(OH)_2$, and ●: CoOOH).

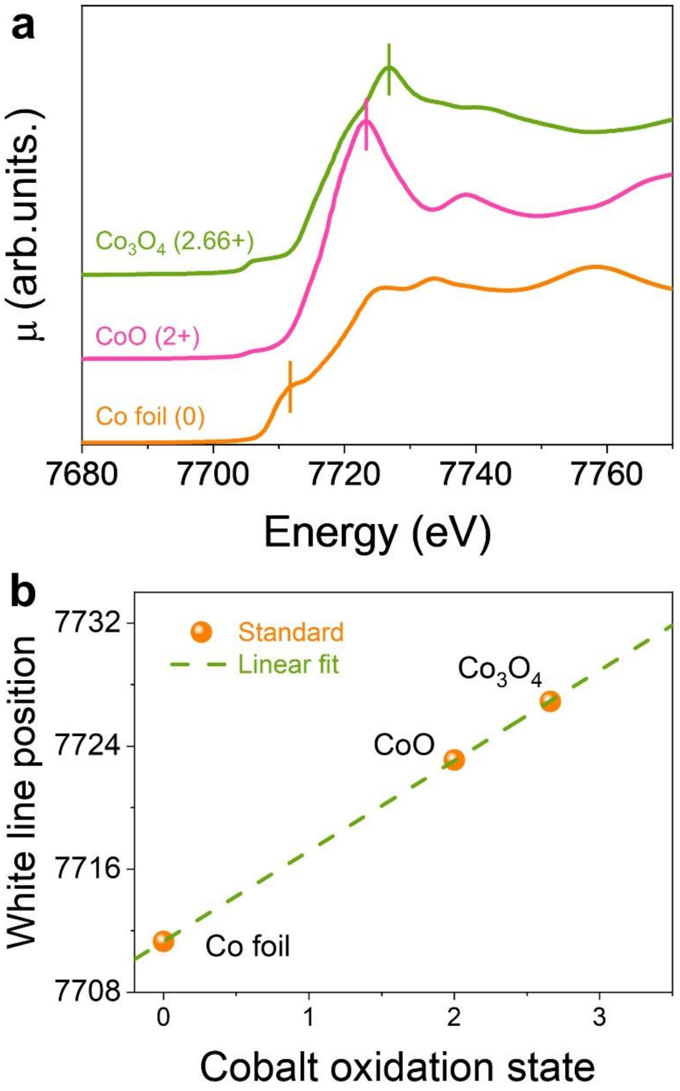

Supplementary Figure 13. **Co K-edge XANES spectrum of Co standard samples.** (a) white line peak. (b) Linear fitting curve of white line position and cobalt oxidation states.

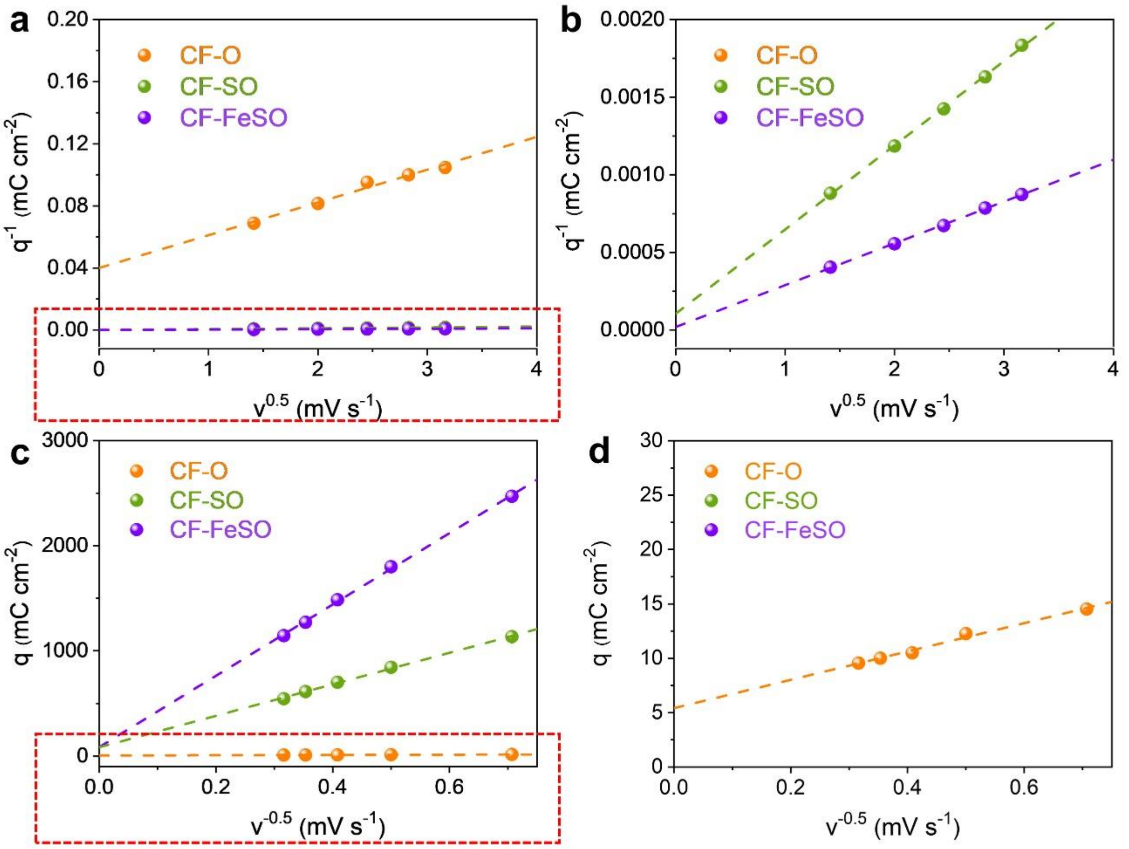

Supplementary Figure 14. **Calculation for internal and external voltametric charge densities.** Linear plot of (a) q^{-1} vs. $v^{0.5}$ and (c) q vs. $v^{-0.5}$ of CF-based (b) and (d) are enlarged graphs of the red dotted boxes in (a) and (c), respectively.

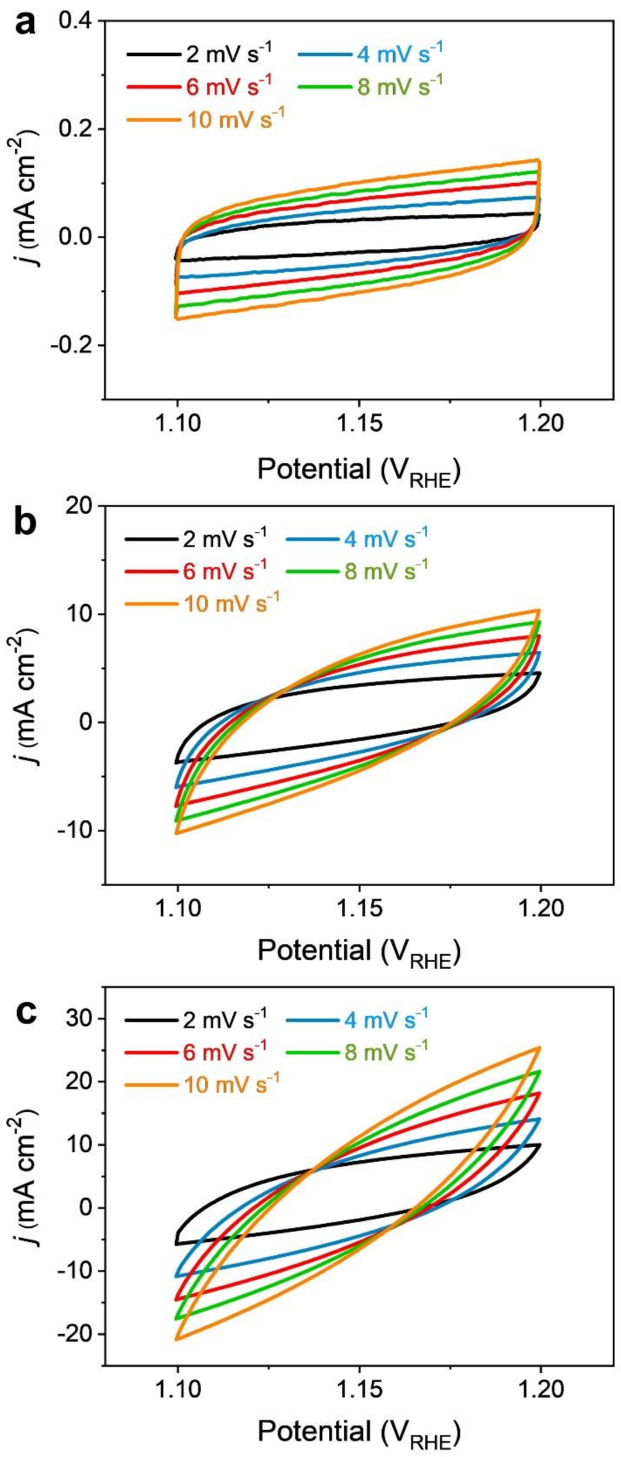

Supplementary Figure 15. **Cyclic voltammetry of Co foam-based electrodes with different scan rate (2, 4, 6, 8 and 10 mV s^{-1}).** (a) CF-O, (b) CF-SO and (c) CF-FeSO.

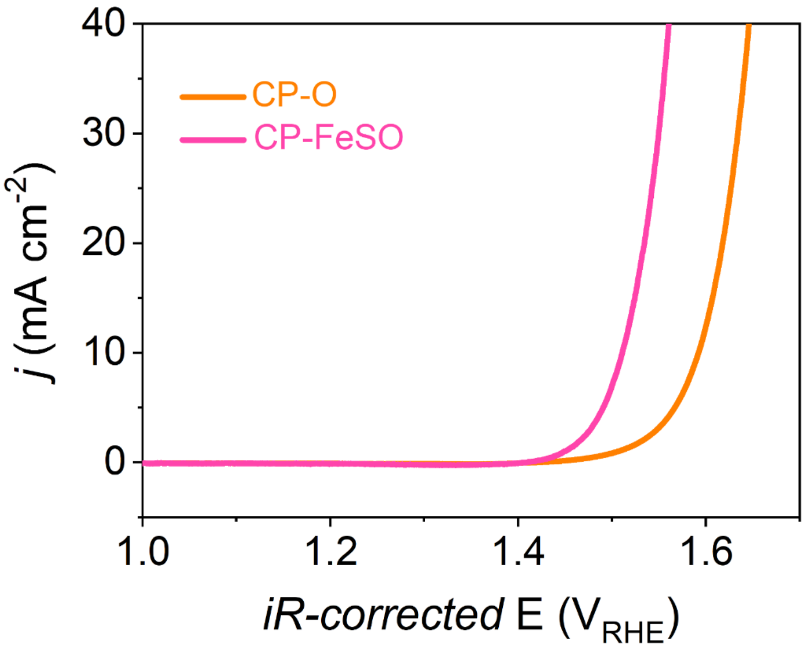

Supplementary Figure 16. Electrocatalytic activity of CP-O and CP-FeSO for OER measured in O_2-saturated 1 M KOH solutions with rotation at 1600 rpm. Catalyst loading: 50 μg cm^{-2}. Working electrode: 0.196 cm^2 glassy carbon.

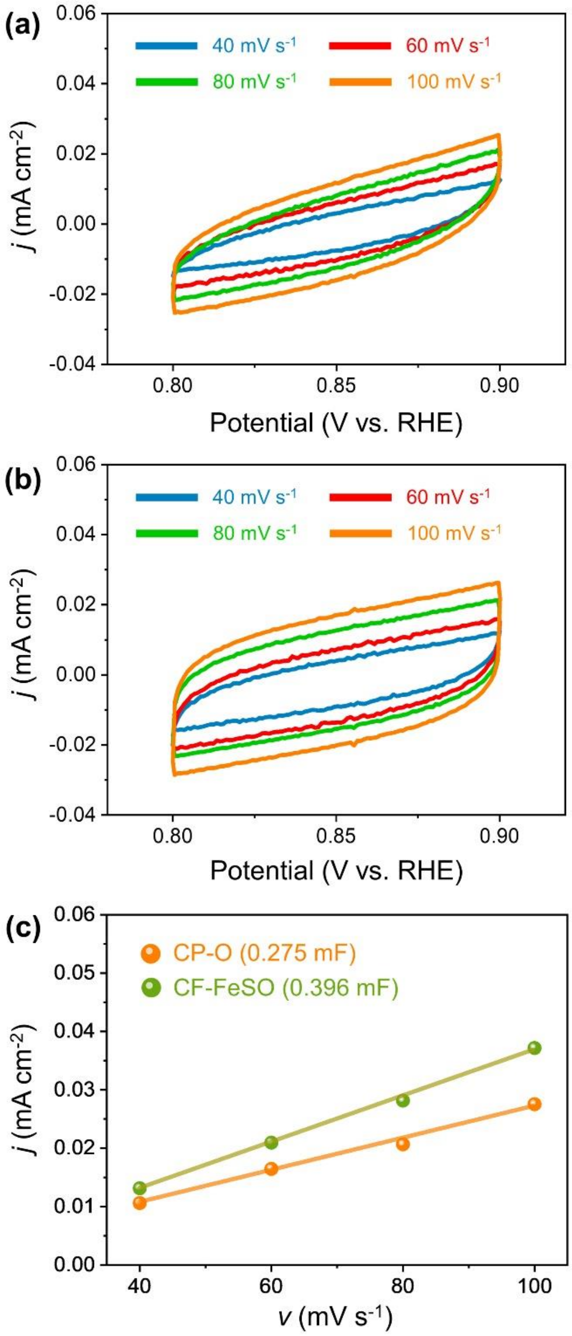

Supplementary Figure 17. Electrochemically active surface areas (ECSA) calculation. Cyclic voltammetry of (a) CP-O and, (b) CP-FeSO in 1 M KOH solution at scan rate of 40, 60, 80 and 100 mV s^{-1}. (c) Capacitive current density versus scan rate CP-O and CP-FeSO to calculate (ECSA. Catalyst loading: 50 μg cm^{-2}. Working electrode: 0.196 cm^2 glassy carbon.

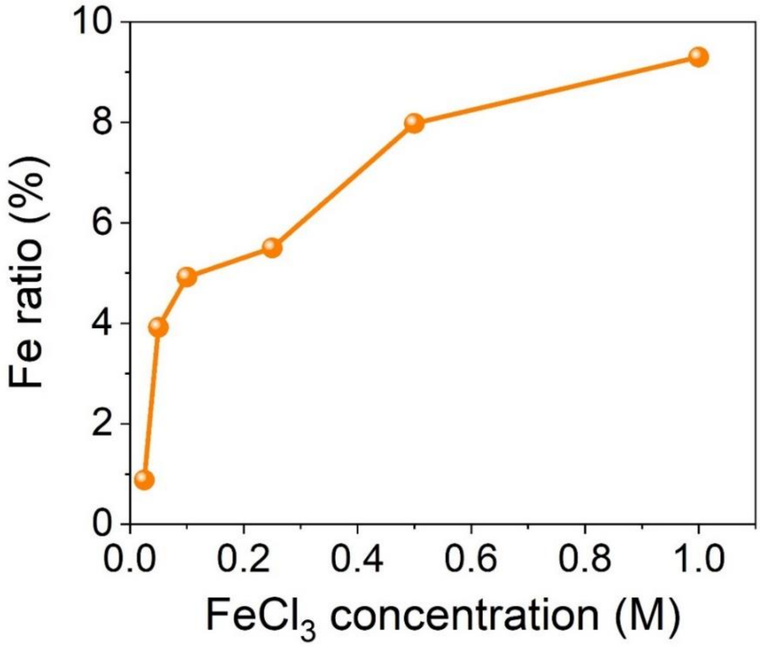

Supplementary Figure 18. The Fe content graph of CF-FeSO electrodes with different $FeCl_3$ concentrations. Fe atomic ratios of CF-FeSO was measured by SEM-based EDS analysis.

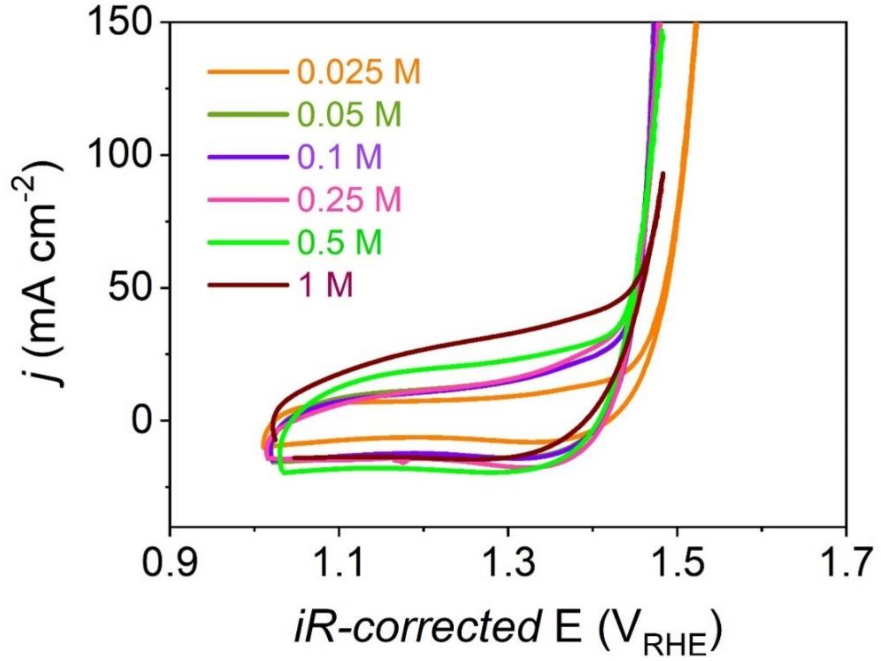

Supplementary Figure 19. **Electrocatalytic OER activity of CF-FeSO for various $FeCl_3$ concentration in the dipping solution.** Cyclic voltammetry (CV) of CF-FeSO with different Fe atomic ratio in 1 M KOH solution.

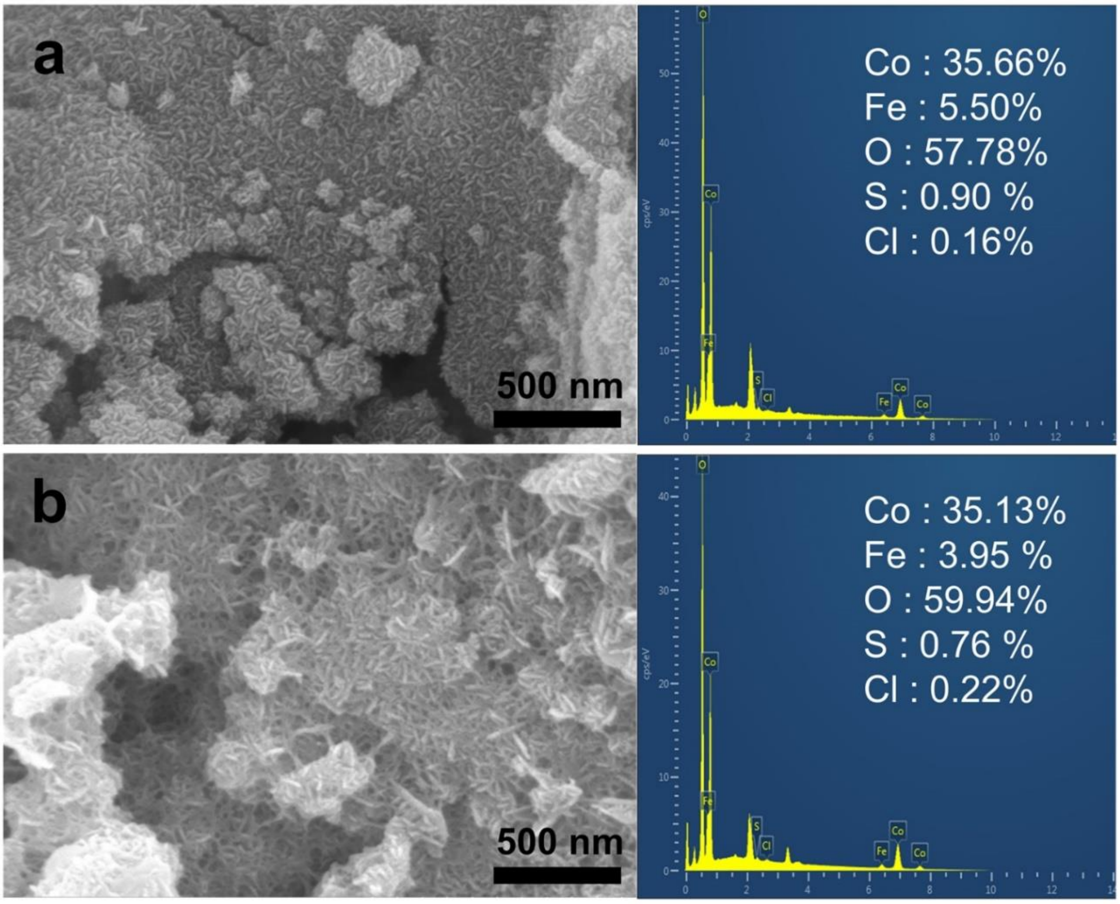

Supplementary Figure 20. **SEM and EDS results of CF-FeSO.** (a) Before and (b) after durability test.

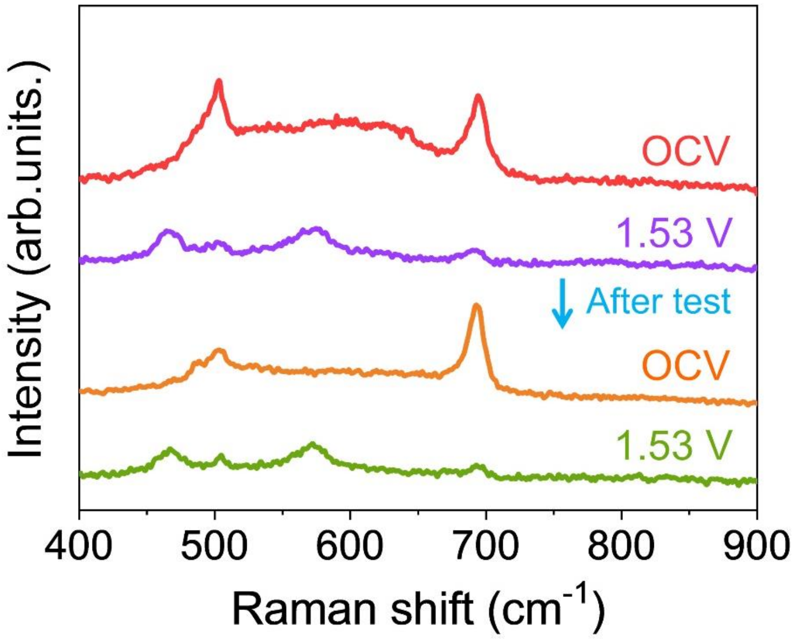

Supplementary Figure 21. ***In-situ/operando*** **Raman spectrum of tested CF-O**. After 1.53 V tests, CF-O electrode was withdrawn from the electrolyte. The tested CF-O electrode was remeasured at OCV and 1.53 V.

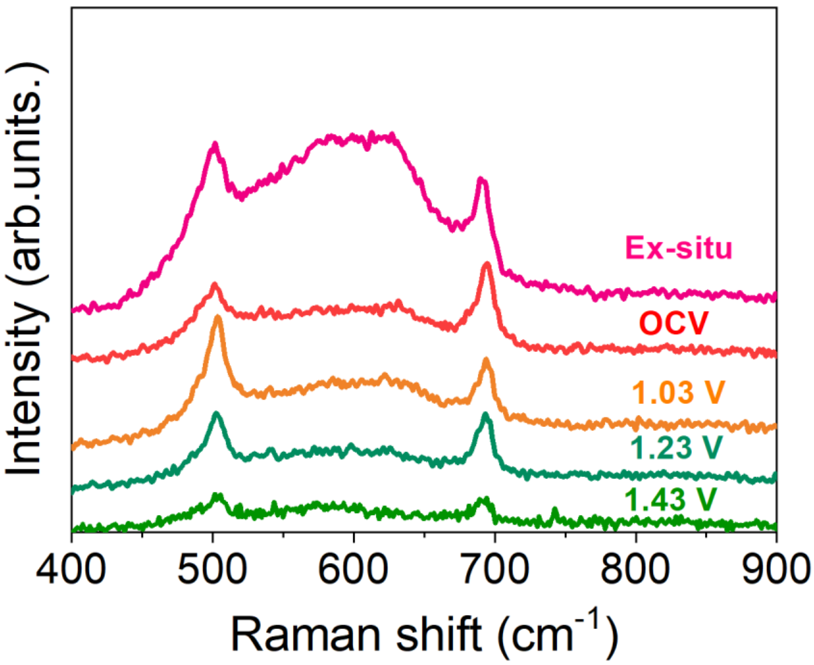

Supplementary Figure 22. ***In-situ/operando*** **Raman spectrum of CF-SO electrode.** The *operando* Raman was measured using hand-made electrochemical flow cell (as shown in Figure 4a). Reference electrode: Ag/AgCl. Counter electrode: Pt wire.

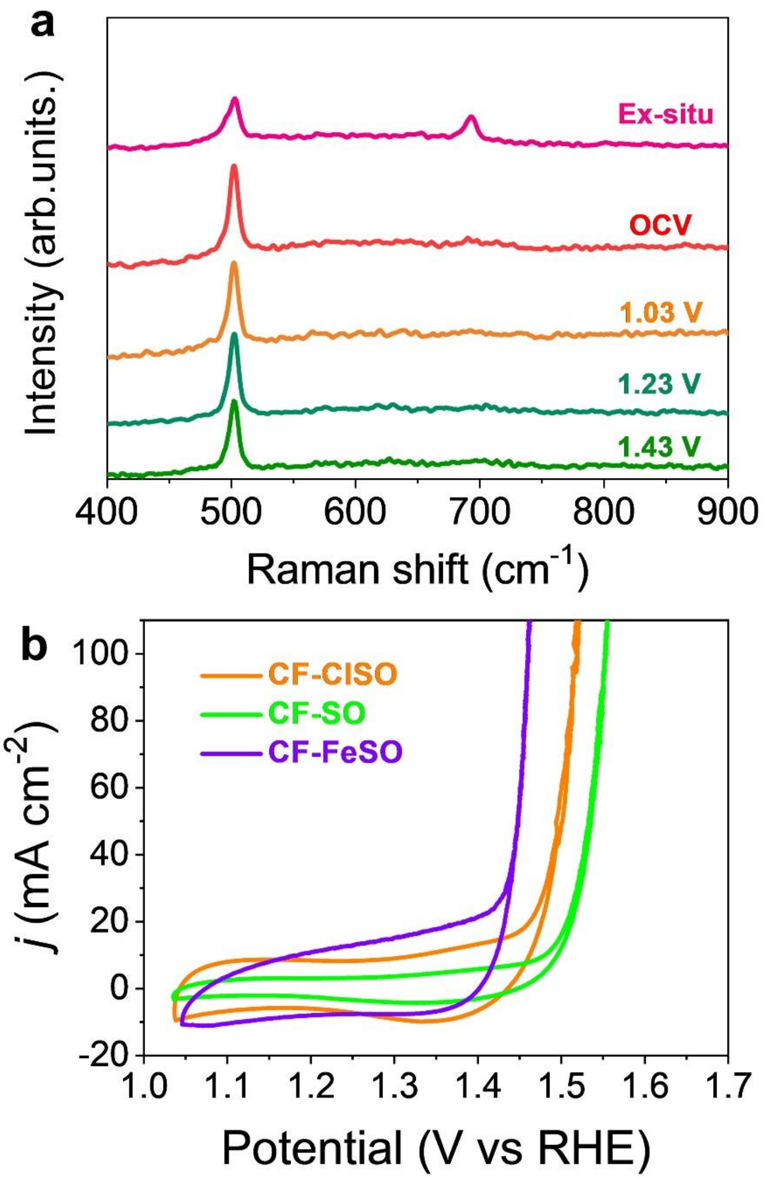

Supplementary Figure 23. Raman spectrum and OER activity of CF-ClSO electrode. (a) *In-situ/operando* Raman spectrum of CF-ClSO electrode. The *operando* Raman was measured using hand-made electrochemical flow cell. (b) Electrocatalytic OER activity of CF-ClSO electrode in 1 M KOH.

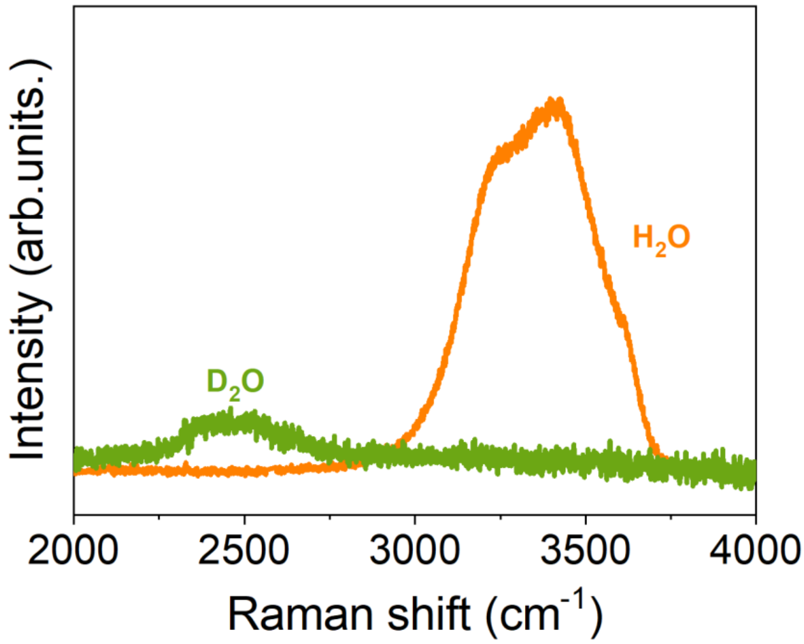

Supplementary Figure 24. ***In-situ/operando*** **Raman spectrum using H_2O and D_2O.** Raman results obtained under hand-made electrochemical flow cell conditions using H_2O and D_2O as electrolytes, respectively.

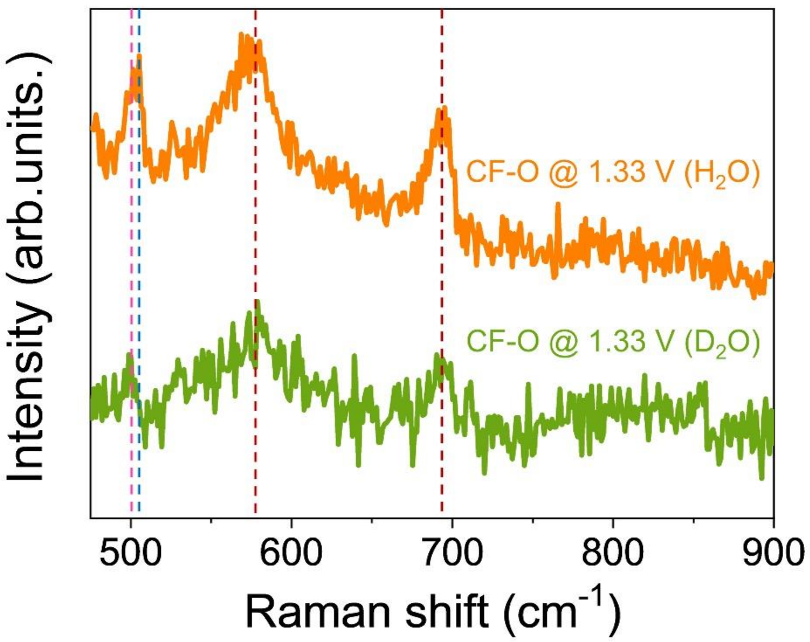

Supplementary Figure 25. ***In-situ/operando*** **Raman spectrum of CF-O electrode at 1.33 V_{RHE} using H_2O and D_2O.** The *operando* Raman was measured using hand-made electrochemical flow cell.

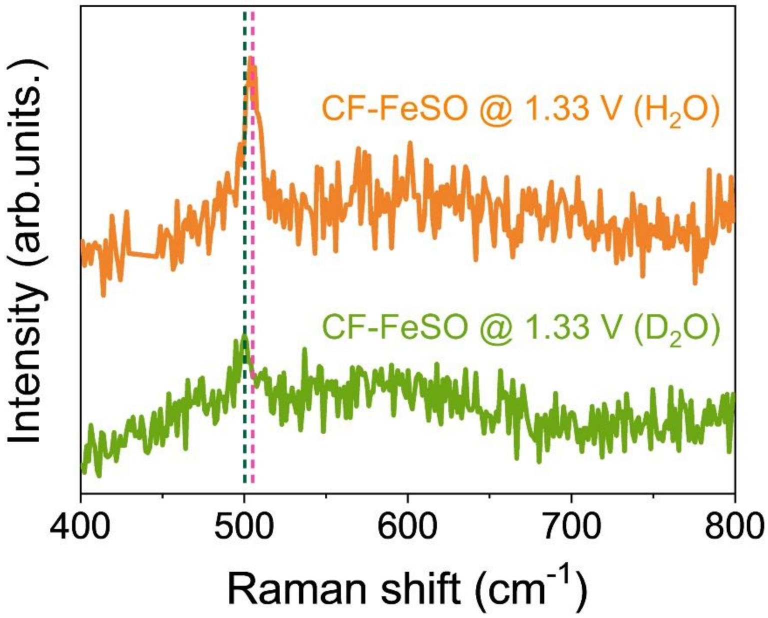

Supplementary Figure 26. ***In-situ/operando*** **Raman spectrum of CF-FeSO electrode at 1.33 V_{RHE} using H_2O and D_2O.** The *operando* Raman was measured using hand-made electrochemical flow cell.

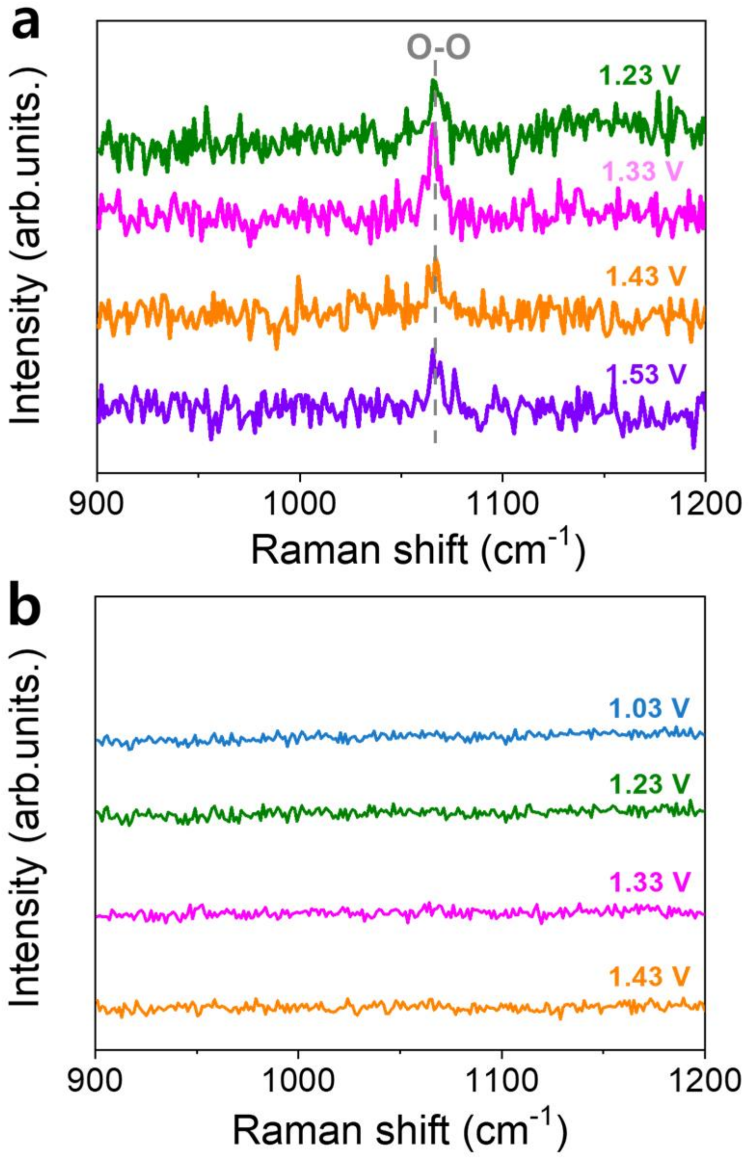

Supplementary Figure 27. *In-situ/operando* Raman spectrum for observing oxygen intermediate. The *operando* Raman was measured using hand-made electrochemical flow cell. (a) CF-O and (b) CF-FeSO.

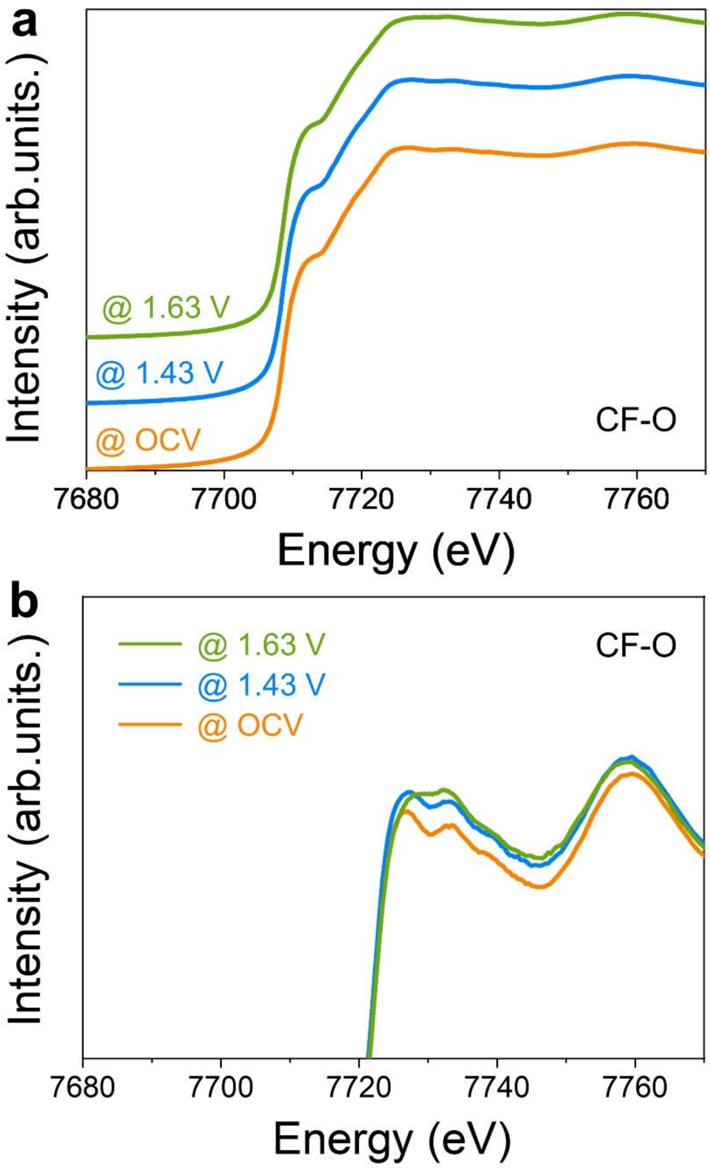

Supplementary Figure 28. (a) *In-situ/operando* Co K-edge XANES spectrum of CF-O electrode and (b) enlarged graph showing XANES peak. The *operando* X-ray absorption spectroscopy (XAS) was measured using hand-made electrochemical flow cell. XAS was conducted at the 1D beamline of the Pohang Accelerator Laboratory (PAL), Pohang, South Korea.

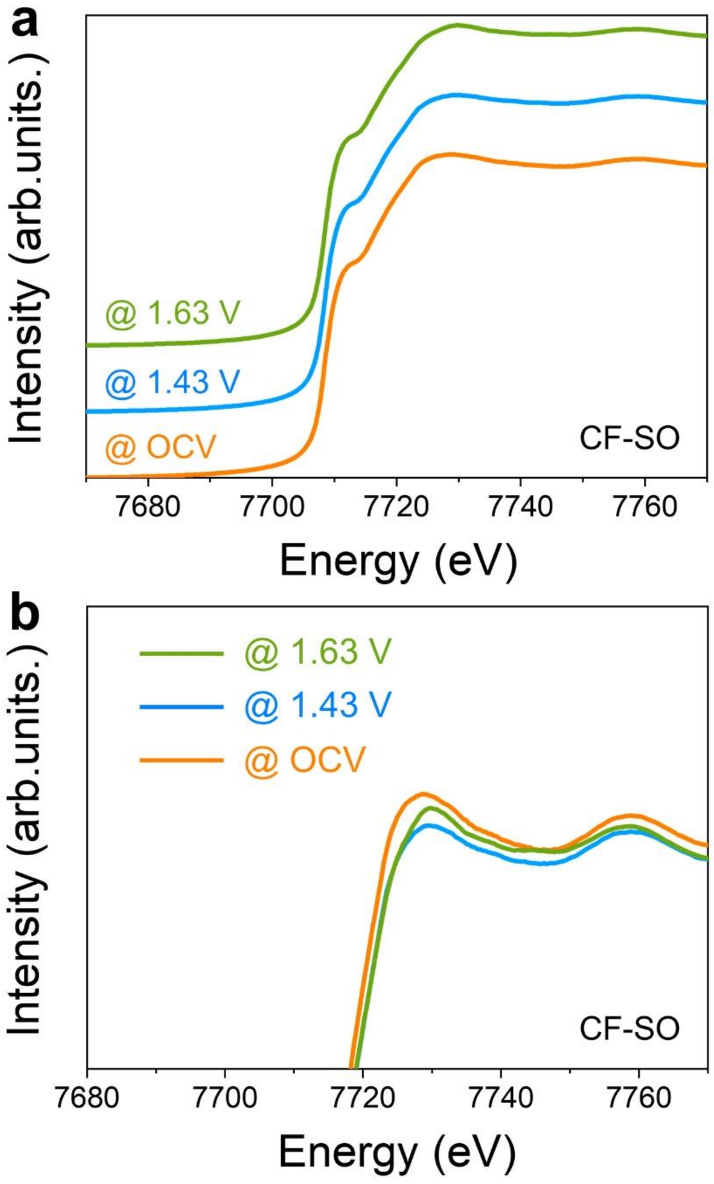

Supplementary Figure 29. (a) *In-situ/operando* Co K-edge XANES spectrum of CF-SO electrode and (b) enlarged graph showing XANES peak. The *operando* X-ray absorption spectroscopy (XAS) was measured using hand-made electrochemical flow cell. XAS was conducted at the 1D beamline of the Pohang Accelerator Laboratory (PAL), Pohang, South Korea.

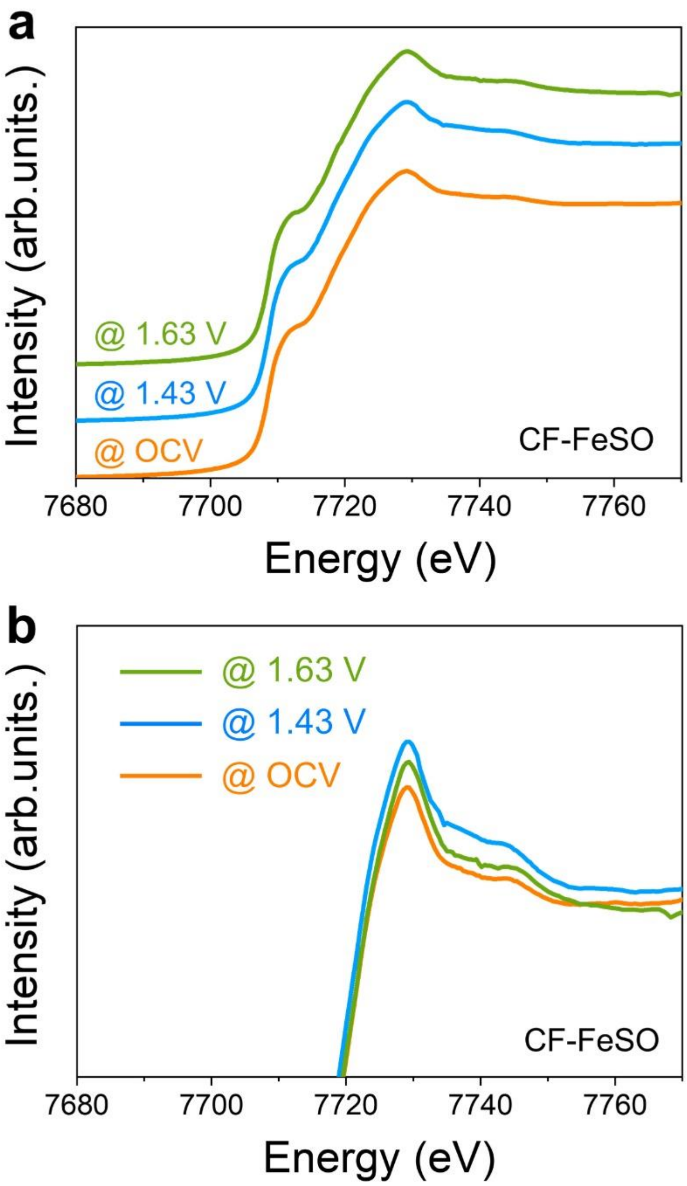

Supplementary Figure 30. (a) *In-situ/operando* Co K-edge XANES spectrum of CF-FeSO electrode and (b) enlarged graph showing XANES peak. The *operando* X-ray absorption spectroscopy (XAS) was measured using hand-made electrochemical flow cell. XAS was conducted at the 1D beamline of the Pohang Accelerator Laboratory (PAL), Pohang, South Korea.

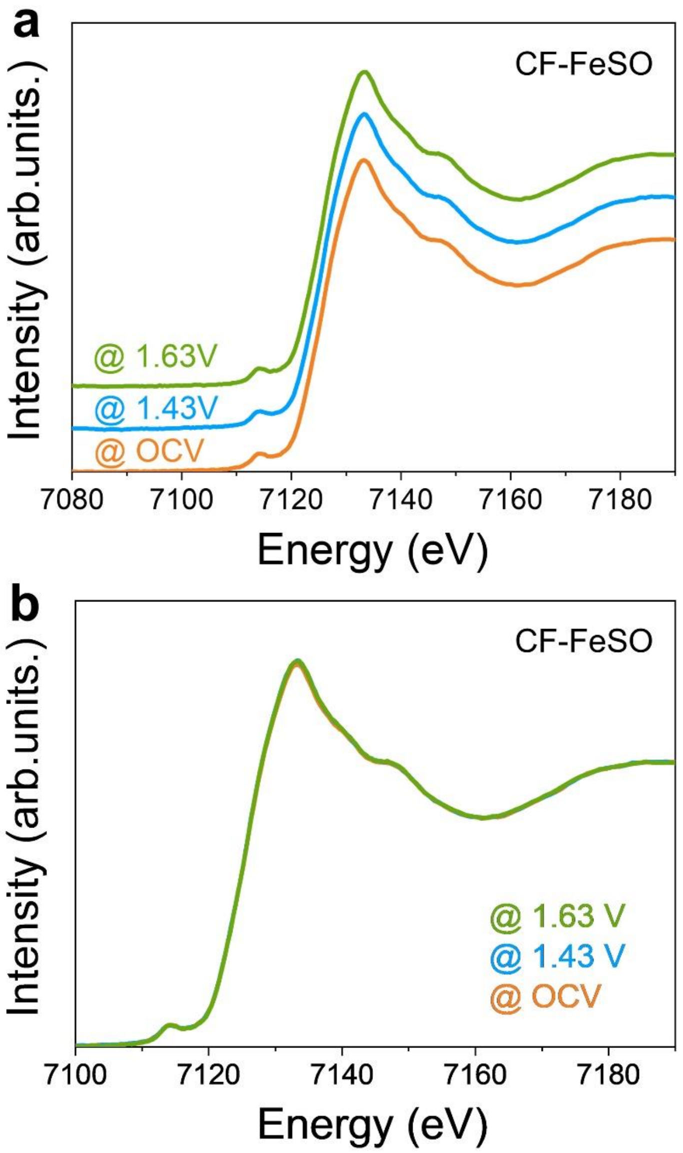

Supplementary Figure 31. (a) *In-situ/operando* Fe K-edge XANES spectrum of CF-FeSO electrode and (b) superimposed graph of each XANES. The *operando* X-ray absorption spectroscopy (XAS) was measured using hand-made electrochemical flow cell. XAS was conducted at the 1D beamline of the Pohang Accelerator Laboratory (PAL), Pohang, South Korea.

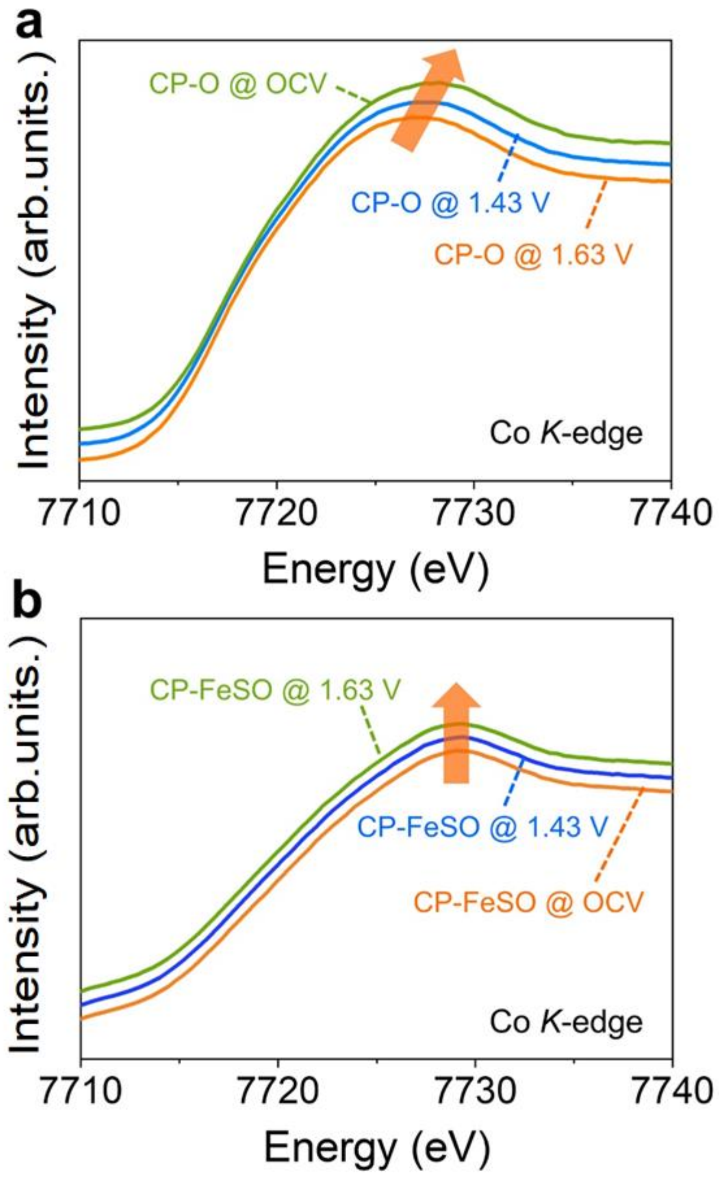

Supplementary Figure 32. *In-situ/operando* Co K-edge XANES spectrum for powder type. The *operando* X-ray absorption spectroscopy (XAS) was measured using hand-made electrochemical flow cell. XAS was conducted at the 1D beamline of the Pohang Accelerator Laboratory (PAL), Pohang, South Korea. (a) CP-O and (b) CP-FeSO.

Supplementary Note 1. ***In-situ/operando analysis techniques***

- ***X-ray absorption spectroscopy (XAS)***

In-situ/operando XAS studies were conducted using custom-made electrochemical flow cell in fluorescence mode at 1D beamline of Pohang Accelerator Laboratory (PAL), Pohang, South Korea. Ag/AgCl (3 M NaCl) and Pt wire were used as reference electrode and counter electrode, respectively. Synthesized Co foam (CF) electrode was used for working electrode and attached to carbon coated kepton film, which is on the beam position. To remove oxygen burble, the electrolyte is flowed by peristaltic pump. The XAS data was collected at an angle of 45° with respect to beam line and detector. The detail setting was reported in previous paper.[1] The data analysis was conducted using Athena software.

- **Raman spectroscopy**

In-situ/operando Raman spectroscopy was performed in standard three-electrode system using CF based electrode as the working electrode, a platinum wire as the counter electrode and Hg/HgO electrode as the reference electrode. IVIUM CompactStat was used as a potentiostat. Laser wavelength of 785 nm was used. The scheme of cell for Raman spectroscopy is illustrated in **Figure 4a.** 1 M KOH was used as electrolyte. The thin electrolyte layer covered with CF based electrode to observe surface of electrode by Raman spectroscopy.

- ***NEXAFS***

Soft X-ray based near edge X-ray adsorption fine structure (NEXAFS) measurements at Co L-edge and O K edge were conducted at the 10D KIST beamline of the Pohang Accelerator Laboratory. To measure the *in-situ/operando* NEXAFS, homemade electrochemical cell was manufactured using PEEK. The scheme of cell for NEXAFS is illustrated in **Figure 4b**. To making electronic contact, 20 nm Au and 10 nm Ti was coated on 100 nm SiN window (1 mm × 1mm window size, Norcada). The Catalyst was loaded on Au coated SiN window by drop casting method. Catalyst coated SiN window was used as working electrode. Rh wire and Ag wire were used as counter and reference electrode, respectively. The 1 M KOH was used as electrolyte. The data was corrected using silicon drift detector (SDD) at fluorescence yield mode.

Supplementary Note 2. ***Calculation of internal and external voltammetric charge densities and the electrochemical porosities***

Trasatti et al. (main manuscript Ref. 48) proposed this electrochemical calculation process.[2]

Total voltammetric charge (q_t^*) : $q_t^* = q_i^* + q_e^*$ (1)

(q_i^* = internal voltammetric charge , q_e^* = external voltammetric charge)
If the scan rate is infinitely fast, only external voltammetric charge will be measured. When scan rate is infinitely close to zero, internal and external voltammeric charge will be measured together.

$$q^*(\nu) = q_e^* + A\left(\frac{1}{\sqrt{\nu}}\right) \quad (2)$$

$$\frac{1}{q^*(\nu)} = \frac{1}{q_t^*} + B\sqrt{\nu} \quad (3)$$

Based on equation (2) and (3) and CV results at different scan rates (Supplementary Figure 12,13), internal and external voltammetric charge densities can be calculated.
Electrochemical porosity (ϕ) = $\frac{q_i^*}{q_e^*}$
Electrochemical porosity is defined as the ratio of internal voltammetric charge to external voltammetric charge, showing roughness of electrode.

Supplementary References

1. Lee, W. H.; Nong, H. N.; Choi, C. H.; Chae, K. H.; Hwang, Y. J.; Min, B. K.; Strasser, P.; Oh, H.-S., Carbon-Supported $IrCoO_x$ nanoparticles as an efficient and stable OER electrocatalyst for practicable CO_2 electrolysis. *Applied Catalysis B: Environmental* **2020**, 118820.
2. De Pauli, C.; Trasatti, S., Electrochemical surface characterization of IrO_2 + SnO_2 mixed oxide electrocatalysts. *Journal of Electroanalytical Chemistry* **1995,** *396* (1-2), 161-168.
